# Pt NPs Supported on CeO_2_/C as Electrocatalysts for Oxygen Reduction Reaction: Novel Physicochemical Insights on the Synthesis and on the Improved Activity and Stability

**DOI:** 10.1002/smll.202403127

**Published:** 2025-05-05

**Authors:** Mattia Parnigotto, Gregorio Dal Sasso, Enrico Berretti, Marco Mazzucato, Federica Bertolotti, Alessandro Lavacchi, Maria Chiara Dalconi, Luca Gavioli, Christian Durante

**Affiliations:** ^1^ Department of Chemical Sciences University of Padova Via Marzolo 1 Padova 35131 Italy; ^2^ Italian National Research Council Institute of Geosciences and Earth Resources Corso Stati Uniti 4 Padova 35121 Italy; ^3^ Italian National Research Council Institute of Chemistry of Organometallic Compounds CNR‐ICCOM Via Madonna del Piano 10 Sesto Fiorentino 50019 Florence Italy; ^4^ National Interuniversity Consortium of Materials Science and Technology INSTM Via G. Giusti 9 Florence 50121 Italy; ^5^ Department of Science and High Technology and To.Sca.Lab University of Insubria Via Valleggio 11 Como 22100 Italy; ^6^ Department of Geosciences University of Padova Via Gradenigo 6 Padova 35131 Italy; ^7^ Department of Mathematics and Physics Università Cattolica del Sacro Cuore via della Garzetta 46 Brescia 25133 Italy

**Keywords:** CeO_2_, gas diffusion electrode (GDE), oxygen reduction reaction (ORR), proton exchange membrane fuel cell (PEMFC), Pt nanoparticles (Pt NCs), wide‐angle X‐ray total scattering (WAXTS)

## Abstract

This study emphasizes the effect of CeO_2_ on the Pt nanoparticle (NP) dimension, stability, and activity versus the oxygen reduction reaction. It is demonstrated that the one‐pot synthesis of Pt NPs along with CeO_2_ NPs over carbon support produces small Pt NPs (2 nm) with higher activity, than the sole Pt NPs, thanks to the cooperative interaction exerted by CeO_2_. This is nicely demonstrated by using synchrotron wide‐angle X‐ray total scattering and advanced data analysis, monitoring the in situ nucleation and growth of Pt NPs in the presence of preformed CeO_2_ NPs or of a Ce precursor. Raman, X‐ray photoelectron spectroscopy, and high‐resolution transmission electron microscopy analyses are carried out to support the formation of oxygen vacancies responsible for the metal–support interaction. Moreover, the most effective catalyst, PtCeO_2_/C250 (mass activity: MA_0.9 V_ = 423 Ag^−1^; specific activity: SA_0.9 V_ = 446 µAcm^−2^), exhibits activity comparable to the commercial benchmark Pt/C, yet significantly greater stability as demonstrated by accelerated stress tests conducted on gas diffusion electrode. Specifically, PtCeO_2_/C250 retains 62% ± 7% of its MA_0.65 V_ and 79% ± 9% of its SA_0.65 V_, compared to 43% ± 5% and 62% ± 7%, respectively, for the benchmark.

## Graphical abstract

The effect of CeO_2_ on the Pt nanoparticles nucleation and growth and on the activity versus the oxygen reduction reaction was studied by in situ synchrotron wide‐angle X‐ray total scattering. The most active catalyst (MA = 423 A g^−1^; SA = 446 µA cm^−2^) shows greater stability than the commercial benchmark as demonstrated by accelerated stress tests at gas diffusion electrode.

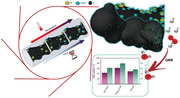

## 1 Introduction

Stable platinum‐based materials are essential for developing highly durable proton exchange membrane fuel cells (PEMFCs), especially if the end‐user is in the automotive sector. In this regard, the United States Department of Energy has set a target for light‐duty vehicles of 5000 h by 2025 and 8000 h by 2030.^[^ ^1^ ^]^ Currently, durability stands at ≈4000 h, and most of the gap is expected to be bridged by improvements in cathodic and anodic catalyst stability.^[^ ^1^ ^]^ This is particularly true for the cathode side of a PEMFC where most of the Pt is employed.

Although platinum catalysts are highly active toward the oxygen reduction reaction (ORR), their insufficient durability slows down the reliability for widespread commercialization. Platinum catalysts undergo structural and morphological changes during the operating conditions of PEMFC, which result in a loss of performance over time. According to the Pt Pourbaix diagram the tendency of Pt to dissolve occurs as electrode potential is between 0.92 and 1.10 V (relative to standard hydrogen electrode), which corresponds to the typical working potential of the cathode of PEMFCs. This depends on the Pt^2+^ concentration, and other operative parameters, that can enlarge or shrink the thermodynamic instability region, but as rough estimation is generally valid. In addition, the size of the Pt NPs also affects its dissolution potential, which inversely scales with the particle radius as for the Gibbs–Thomson equation, so that smaller NPs are oxidized at less positive potential.^[^ ^2^ ^]^ From the kinetics point of view, the thermodynamic constraints are loosened, however, this is not sufficient to guarantee a large potential window of stability. Pt NPs degradation entails different mechanisms, which usually occur simultaneously: i) dissolution of the metal component and subsequent redeposition on more stable NPs, ii) Ostwald ripening, iii) agglomeration of small NPs, iv) particle detachment, and v) support degradation.^[^ ^3^, ^4^ ^]^ Concerning the metal dissolution it is well‐established that platinum undergoes a stepwise oxidation process ending up with the formation of PtO_2_, and both the metal and the oxide are subject to dissolution reactions.^[^ ^5^ ^]^ The Pt NPs support, typically carbon‐based, can also undergo degradation, which in this specific case involves carbons oxidation to CO_2_.^[^ ^6^ ^]^

The two main approaches for addressing the problem of stability are to play with the particle composition and the Pt NPs support. Alloying platinum with transition metals reduces the costs of the catalyst because of a reduced load of Pt and can boost the ORR.^[^ ^7^ ^]^ Alloying can modify the dissolution potential of Pt NPs, even if this change is only of a few millivolts, so negligible from the applicative point of view. On the other hand, the alloying with non‐noble metal elements (such as Fe, Co, Ni, and rare earth elements), to form Pt‐based alloys can reduce the kinetic stability of the particle since transition metals are more likely to be oxidized and dissolved, working as sacrificial element preserving Pt dissolution. This effect is not necessarily detrimental, as the alloying effect may be indeed lost, but the resulting carved dealloyed particles are typically still active due to strain effects.^[^ ^8^ ^]^ However, in the long run, this results in decreased durability under proton exchange membrane (PEM) fuel cell working conditions, which hinders their practical application.^[^ ^3^ ^]^

Ostwald ripening, agglomeration, aggregation, and coalescence are very often the result of support corrosion. In fact, preferential local corrosion of the support near NPs could ease their migration due to the shrinkage of the support surface. When corrosion leads to a very weak interaction between the NPs and carbon support, particle detachment may occur. Ultimately, severe carbon support corrosion could lead to a loss of structural integrity deteriorating the porous channels, which are vital for reactant transport, and dramatically decreasing electrical conductivity, essential to efficiently close the fuel cell circuit.^[^ ^9^ ^]^ To enhance the ability to immobilize particles on the carbon support, various approaches have been explored, including the doping of carbon support with heteroatoms such as nitrogen and sulfur.^[^ ^10^, ^11^, ^12^, ^13^ ^]^ This can induce specific chemical and electronic interactions with the metal NPs, thereby reducing the mobility of the NPs and, consequently, their degradation. The hierarchical porous structure can also play a critical role in confining Pt NPs and reducing leaching or detachment.^[^ ^10^ ^]^

In comparison to highly graphitized carbon materials, non‐carbon supports have received enormous attention in the last decade since they can completely avoid electrochemical corrosion during PEM fuel cell long‐term operation and repeated start/stop cycles.^[^ ^14^, ^15^ ^]^ Among all the commercially available oxides, ceria (CeO_2_) is one of the most used compounds in industry, from mechanical processing to catalysis.^[^ ^16^, ^17^ ^]^ The widespread utilization in catalysis is related to the reversible Ce^4+^/Ce^3+^ redox couple, which originates because of its intrinsic defective structure.^[^ ^18^ ^]^ Pure stoichiometric ceria shows a fluoritic structure where atoms are arranged in a face‐centered cubic cell. The non stoichiometric ceria (CeO_2−δ_) is generated by the release of oxygen and consequently the formation of oxygen vacancy inside the crystal structure according to the following mechanism

(1) $$\mathrm{Ce}O_{2}\to \;\mathrm{Ce}O_{2-\delta }+\frac{\delta }{2}O_{2}\left(g\right)$$

The vacancy formation leaves two electrons localized into two cerium cations, thus altering the oxidation state from Ce^4+^ to Ce^3+^. As the size of Ce^3+^ is larger than Ce^4+^, a lattice strain will be introduced in its neighborhood, thereby affecting the local charge distribution. DFT calculations have proven that the formation of vacancies is energetically more favored at the nanoscale rather than in bulk ceria.^[^ ^19^ ^]^ For this reason, ceria nanosizing has triggered innumerable applications in catalysis. Notwithstanding, the capability to control the Ce^4+^/ Ce^3+^ ratio, which is of high importance in catalysis, remains a challenge.

Ceria is incorporated into the PEM fuel cells of the Toyota 2017 Mirai, since Pt/CeO*_x_* composite material shows an exceptionally high activity for the hydrogen oxidation reaction and high durability, compared to standard Pt/C catalysts. Ceria can also act as a radical scavenger during the device's operating conditions, preserving the Nafion membrane from hydroxyl radicals.^[^ ^20^ ^]^ CeO_2_ has also been proposed as an additive for the catalysts layer of the cathodic side of the PEMFC. It has been demonstrated that CeO_2_ impacts the stability of the catalyst, as it avoids the sintering of platinum NPs. In addition, it has been reported that the interaction between Pt and CeO_2_ can increase the activity for ORR compared to Pt/C and this is attributed to the high oxygen storage capacity of ceria and the oxygen spillover from CeO_2_ to Pt.^[^ ^21^, ^22^, ^23^, ^24^ ^]^ This is why understanding how to produce Pt NPs supported on a CeO_2_/carbon composite material is strategic, as it unveils the physicochemical properties underlying the electrocatalytic behavior. In this regard, the recent literature proposes complex two‐step synthesis (where Pt and ceria are synthesized in two different steps and with two different methods),^[^ ^25^, ^26^ ^]^ or one‐pot synthesis via a solvothermal method.^[^ ^27^, ^28^ ^]^

In this study, the focus is on developing a novel, competitive, and straightforward synthesis method for Pt NPs on a composite ceria–carbon Vulcan XC72 support (PtCeO_2_/C) and exploring the stability benefits of ceria NPs in the cathodic compartment by easy to setup and reliable half‐cell GDE tests. An easy and safe one‐pot (ceria and Pt) solid‐state synthesis is proposed and deeply studied through a specifically designed reactor for in situ synchrotron X‐ray diffraction measurements. This approach facilitated the monitoring of the growth of both ceria and Pt NPs during the synthesis process. The in situ characterization of PtCeO_2_/C catalysts has been carried out through synchrotron wide‐angle X‐ray total scattering (WAXTS) experiments coupled to advanced data analysis in the reciprocal space through the Debye scattering equation (DSE) method.^[^ ^29^, ^30^ ^]^ The DSE analysis of WAXTS data, based on atomistic models, provides statistically robust measures of structural and microstructural properties based on a unified modeling approach, obtaining a quantitative description of the NPs atomic‐ and nanometer‐scale features such as size, shape, lattice strain, and structural defectiveness. Then, the effect of ceria addition on the catalyst activity and stability is assessed using accelerated degradation tests (ADTs) in an innovative gas diffusion electrode cell, as proposed by Arenz and co‐workers.^[^ ^31^ ^]^

## 2 Results and Discussion

### 2.1 PtCeO_2_/C Synthesis

**Figure 1:**
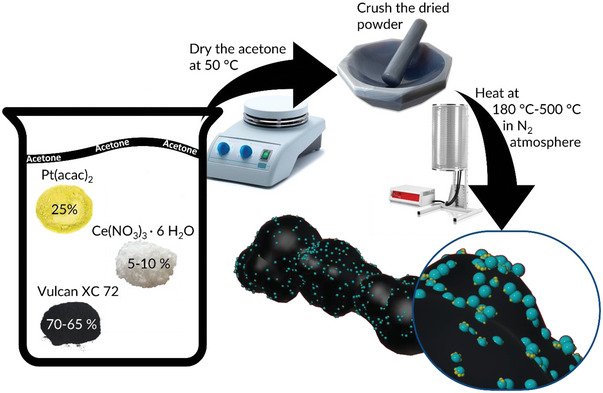
Scheme of the one‐pot solid‐state synthesis: yellow and turquoise NPs are intended for Pt and CeO_2_, respectively.

PtCeO_2_/C was prepared from Pt(acac)_2_ and Ce(NO_3_)_3_ hexahydrate as the precursors for Pt and Ce, respectively. Vulcan was adopted as carbon support. The synthesis was optimized by considering different temperatures in the range of 180–500 °C and to obtain a final CeO_2_ weight percentage of 5%; a detailed description is reported in the experimental part and a sketch of the steps is depicted in **Figure** **1**. In brief, the mixture of metal precursors and carbon Vulcan XC72 was treated in a tubular furnace for 3 h at five different temperatures: 180, 250, 300, 400, and 500 °C; the resulting samples were named accordingly (for example PtCeO_2_/C500). It is worth stressing that Pt NPs form, even at the lower temperature (180 °C) under the effect of the heating and without the presence of hydrogen as a reducing agent, being Ce^3+^ capable of playing as a reducing species. In this way, safety issues related to H_2_ gas handling can be avoided. To have a clearer picture of the ceria effect, three further samples were prepared at 250 °C: one in the absence of Ce(NO_3_)_3_, which serves as a control experiment, and was named Pt/C250; one more with a Ce(NO_3_)_3_ content to obtain a final ceria content of 10% and named PtCeO_2_(10%)/C250 and the final one using the 5% of commercial ceria NPs (10 nm) and named PtCeO_2_(com)/C250.

### 2.2 Synchrotron Small‐Angle X‐Ray Scattering (SAXS) and WAXTS Analysis

SAXS and WAXTS analyses were adopted to investigate the nucleation and growth of Pt NPs, and thus to get insights into the reciprocal interplay between Pt and CeO_2_ NPs during the synthesis. The X‐ray total scattering approach is particularly suitable for nanomaterials characterization as both the Bragg and diffuse scattering are treated equally and modeled, providing quantitative structural information at the atomic and nanometer length scales on nanostructured and disordered materials.^[^ ^30^, ^32^, ^33^ ^]^

**Figure 2:**
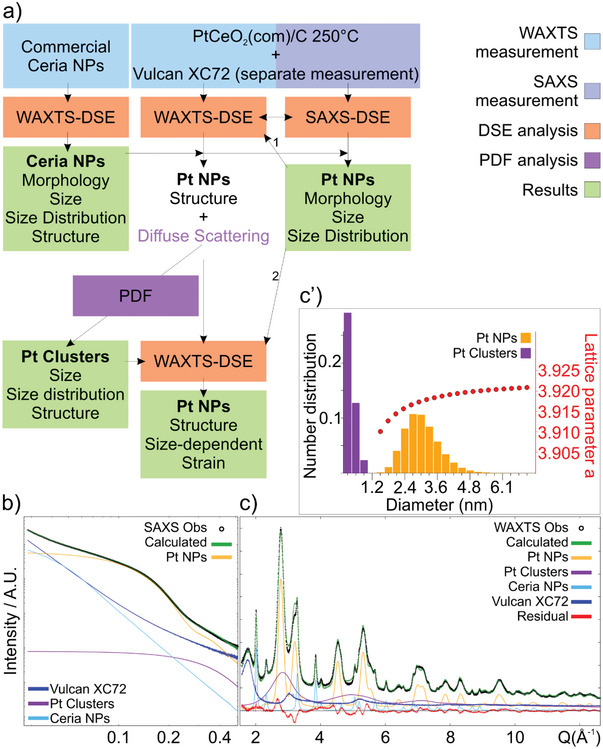
Data of PtCeO_2_(com)/C250 sample. a) Chart flow for the combined SAXS/WAXTS‐DSE analysis. b) SAXS data and best DSE fit (green). SAXS signals of each model component resulting from the SAXS‐DSE analysis, together with the Vulcan XC72 experimental pattern, are also displayed. c) WAXTS data and best DSE fit (green). WAXTS traces of each model component resulting from the WAXTS‐DSE analysis, together with the Vulcan XC72 experimental pattern, are also displayed. c′) Number‐based size distribution of Pt NPs and Pt clusters resulting from the combined DSE analysis of SAXS and WAXTS data is reported, together with the size‐dependent lattice strain (red) refined for Pt NPs.

At first, the PtCeO_2_(com)/C250 catalyst prepared using commercial ceria NPs, has been fully characterized by implementing a recently developed multiscale approach, combining SAXS and WAXTS analyses (**Figure** **2**a), that has been successfully applied to benchmark synthetic, biogenic/biomimetic, and engineered nanomaterials.^[^ ^34^, ^35^, ^36^, ^37^ ^]^

The total scattering pattern in both the small‐ and wide‐angle regions can be computed through the DSE from atomistic models, accounting for the size and shape of NPs as well as for their structure and defectiveness, thus simultaneously providing multiscale information within a unified modeling approach. Even though the SAXS signal is not sensitive to the atomic scale features, the DSE enables the computation of the SAXS pattern from atomistic models, providing information on the size and morphology of NPs. The DSE modeling does not account for particle–particle interactions so it can be reasonably applied when aggregation and concentration effects are negligible. Despite the high computational costs, the DSE modeling of SAXS data has the great advantage that the same size discretization and distribution law of atomistic models can be used to compute both the SAXS and WAXTS patterns enabling the prompt transfer of information on optimized parameters from the small to the wide angle region, i.e., once provided the size and size dispersion of NPs other structural parameters can be safely refined in the DSE analysis of WAXTS data. Thus, the combined analysis of SAXS and WAXTS data, under a unified modeling approach through the DSE, enables a detailed description of NPs from the atomic to the nanoscale.

A separate WAXTS measurement of the commercial ceria NPs was performed to obtain a detailed description of the structure, size, and morphology of NPs (see the Supporting Information for the description of models and results). The DSE‐based analysis provided evidence of a slight morphological anisotropy, also observed in other case studies,^[^ ^38^, ^39^ ^]^ that has been accounted for using a bivariate size distribution model for ceria NPs. The computed X‐ray pattern was optimized against the experimental data by adjusting the lattice parameters, the average size and size dispersion of NPs, and the isotropic atomic displacement parameters. Results show the occurrence of a population of ceria NPs characterized by an average size of ≈8 nm with a significant size dispersion according to a bivariate lognormal size distribution (compatible with the nominal size of 10 nm), and a moderate anisotropic morphology with an aspect ratio (*L*_c_/*D*_ab_) between the two growth directions AR = 1.12 (Figure S1, Supporting Information). Results in Table S1 of the Supporting Information have been promptly transferred in the DSE analysis of PtCeO_2_(com)/C250 catalysts prepared with commercial ceria NPs.

Two measurements were performed on the PtCeO_2_(com)/C250 sample using two operational wavelengths (1.549188 and 0.563291 Å) obtaining the X‐ray total scattering pattern in the small‐ and wide‐angle regions, respectively (Figure 2b,c). The computation of the DSE in the low‐Q range to model the SAXS data complements the morphological characterization of nanomaterials through the DSE modeling at higher Q of WAXTS data.^[^ ^34^, ^35^, ^36^, ^37^, ^40^, ^41^ ^]^ This approach has been fruitfully applied to the characterization of the PtCeO_2_(com)/C250 sample, enabling the use of the same atomistic model to compute both the SAXS and WAXTS patterns, thus simultaneously refining structural and morphological parameters against the two experimental patterns and providing robust quantitative information for a rather complex multiphase composite material. The model has been built based on reasonable assumptions, supported by the available experimental data. Considering that the PtCeO_2_(com)/C250 catalyst has been prepared with commercial ceria, a preliminary DSE analysis of the WAXTS pattern was performed using the results previously obtained on ceria NPs as a model component and accounting for the ceria contribution in the WAXTS pattern of the composite catalyst (Figure 2c). The site occupancy factor of O atoms was refined but no O vacancies were detected. The SAXS and WAXTS patterns of the Vulcan‐XC72, separately measured, were added as a model component to account for the contribution of the carbon support to the total scattering patterns (SAXS and WAXTS) of the PtCeO_2_(com)/C250 sample. Eventually, the DSE signal for Pt NPs was computed from a population of atomistic models of isotropically shaped NPs, generated using a spherical model at increasing size according to one growth direction (diameter *D*), with a diameter step size of 0.3 nm. The Pt f.c.c. unit cell, with cell parameter *a* = 3.9235 Å, was used as a building block for NP construction.

At first, the model used for the DSE‐SAXS analysis of data of PtCeO_2_(com)/C250 included the SAXS experimental pattern of the sole Vulcan‐XC72 and the DSE signal of ceria NPs (Figure 2b), which were scaled to the experimental data, and the DSE signal computed for Pt NPs, for which the average size (*D*) and size dispersion (*σD*), according to a lognormal distribution law, were refined. The best fit of the experimental data was obtained with a population of spherical Pt NPs of average diameter of ≈3 nm and a relative size dispersion of ≈20%. However, the prompt transfer of these results to the wide‐angle region provided a very poor fit, with a significant amount of diffuse scattering that was not fully described (Figure S2a, Supporting Information). When adding a polynomial function as a background component to the model, the fit of the WAXTS pattern significantly improves, revealing the contribution of a very broad, amorphous‐like feature to the whole scattering pattern (Figure S2b, Supporting Information). The atomic pair distribution function (PDF) analysis of the polynomial function was then applied to investigate the nature of the scattering component.^[^ ^42^ ^]^ The *G*(*r*) curve (Figure S2d, Supporting Information) clearly shows a peak at 2.76 Å, compatible with the shortest Pt–Pt distance, providing the possible attribution of the polynomial function to the scattering contribution of sub‐nanometer Pt clusters, as also observed in recently published articles.^[^ ^10^, ^43^ ^]^ To further investigate the occurrence of such Pt clusters, a population of atomistic models of Pt clusters was built, accounting for clusters comprised between Pt_4_ and Pt_75_ and considering the most energetically favored configurations, as reported by Ignatov et al.^[^ ^44^ ^]^ The DSE was computed from these models, providing a scattering trace compatible with the features highlighted by the polynomial function (Figure S2c, Supporting Information). Thus, Pt clusters were added as a further model component together with the scattering pattern of the Vulcan‐XC72 and the DSE signal of ceria NPs and Pt NPs. The occurrence of such Pt clusters is reasonably compatible also with the SAXS signal, as shown by a subtle hump at ≈0.4–0.5 *Q*(Å^−1^) (Figure 2b). However, the relative mass fraction of Pt clusters is highly underestimated in the SAXS‐DSE analysis (5%) with respect to the WAXTS‐DSE one (37%). This could be related to the complex scaling of the signals caused by i) the superimposition of several contributions, considering that most of the characteristic SAXS features are smeared out because of the size polydispersion of NPs;^[^ ^45^ ^]^ ii) the neglection of possible NPs aggregation effects that cannot be modeled by the DSE analysis. On the other hand, the SAXS‐DSE analysis provided robust results on the size and size distribution of Pt NPs, the determination of which was not affected by the uncertainty on the relative mass fraction of Pt NPs and Pt clusters. Thus, the SAXS‐derived model for the size and dispersion of Pt NPs was transferred to the WAXTS‐DSE analysis and those parameters were kept fixed during further refinements. Figure 2c′ reports Pt NPs and Pt clusters diameter distribution and the refined size‐dependent lattice strain (red) for Pt NPs, respectively. The WAXTS‐DSE analysis (Figure 2c) was performed using four model components: the Vulcan‐XC72 scattering trace, the DSE signal of ceria NPs, of Pt clusters, and Pt NPs, the latter with size and size dispersion constrained by the SAXS analysis, whereas a size‐dependent strain has been refined. Results on the average size and number‐ and mass‐based size distribution of Pt NPs were used to calculate the total specific surface area (SSA) of the entire population of Pt NPs, neglecting aggregation effects between NPs. An SSA of 82.8 m^2 ^g^−1^ was calculated by considering Pt NPs of spherical shape at increasing diameter and weighted by their relative number‐based distribution. As it will become clearer later, the obtained result shows good agreement with the electrochemical surface area, as determined by CO stripping. Therefore, the SSA could provide a practical, average estimate of the Pt reactivity enabling the prompt comparison between samples.^[^ ^10^ ^]^ Results of the DSE analysis of SAXS and WAXTS data are collectively reported in **Table** **1**.

**Table 1:** Results from the DSE analysis of the SAXS and WAXTS data of PtCeO_2_(com)/C250. Number‐based average diameter (*D*) and size dispersion (*σD*) of Pt NPs (using a univariate spherical population) and of Pt clusters. Number‐based average size (*D*_ab_, *L*_c_) and size dispersion (*σ*_Dab_, *σ*_Lc_), according to a bivariate size distribution, of ceria NPs, as previously reported. Relative mass fraction of the three model components according to the SAXS and WAXTS‐DSE analysis, specific surface area (SSA) of Pt NPs. Goodness of fit (GoF) of the best DSE fit of SAXS and WAXTS data is also reported.

PtCeO_2_(com)/C250	Pt NPs	Pt clusters	CeO_2_ NPs
*D* [nm] – Number distribution	3.1	0.6	–
*σD* [nm] – Number distribution	0.7	0.2	–
*D*_ab_ [nm] – Number distribution	–	–	7.3
*σD*_ab_ [nm] – Number distribution	–	–	3.2
*L*_c_ [nm] – Number distribution	–	–	8.2
*σL*_c_ [nm] – Number distribution	–	–	2.2
Mass fraction [%] – SAXS	77.0	5.0	18.0
Mass fraction [%] – WAXTS	47.0	37.2	15.8
SSA [m^2 ^g^−1^]	82.8	–	–
GoF SAXS		9.59	
GoF WAXTS		4.41	

### 2.3 In Situ WAXTS Experiments

**Figure 3:**
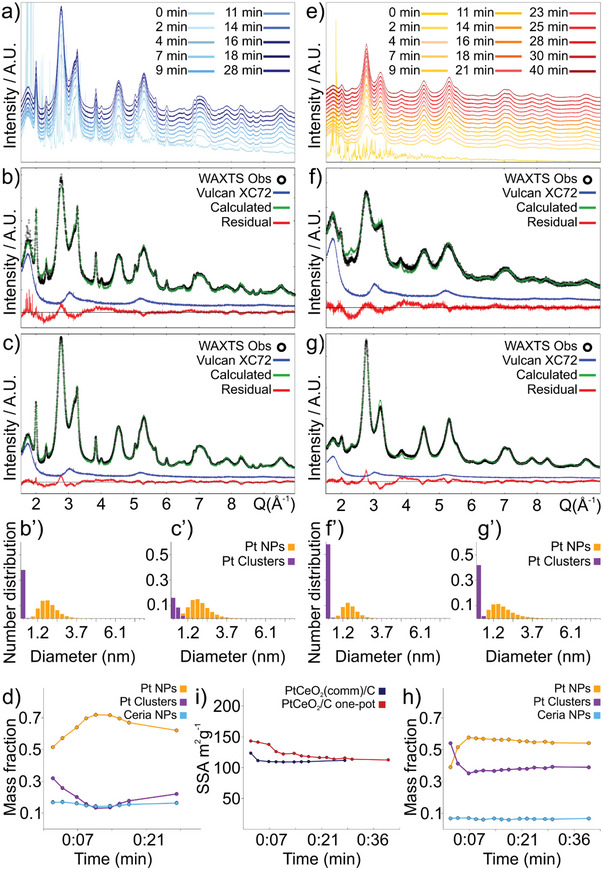
a) In situ WAXTS measurements reproducing the synthesis of PtCeO_2_/C catalyst using commercial ceria; data offset is for display reasons. b,c) Best DSE fit for the first (time = 2 min, b) and the last (time = 28 min, c) WAXTS measurements during reaction since heating. b′,c′) The number‐based size distribution of Pt NPs and Pt clusters resulting from the WAXTS‐DSE analysis of (b) and (c), respectively. d) Relative mass fraction of Pt NPs, Pt clusters, and ceria NPs calculated by the DSE‐WAXTS analysis for each measurement acquired during the reaction. e) In situ WAXTS measurements reproducing the PtCeO_2_/C one‐pot synthesis; data offset is for display reasons. f,g) Best DSE fit for the first (time = 2 min, f) and the last (time = 40 min, g) WAXTS measurements during reaction since heating. f′,g′) The number‐based size distribution of Pt NPs and Pt clusters resulting from the WAXTS‐DSE analysis of (f) and (g), respectively. h) Relative mass fraction of Pt NPs, Pt clusters, and ceria NPs calculated by the DSE‐WAXTS analysis for each measurement acquired during the reaction. i) Total specific surface area (SSA) resulting from the WAXTS‐DSE analysis of the two in situ experiments, calculated taking into account the contribution of Pt NPs weighted for their relative number‐based distribution.

The WAXTS‐DSE analysis used for PtCeO_2_(com)/C250 was extended to enable the in situ investigation of the one‐pot synthesis of PtCeO_2_/C catalyst, to optimize the synthesis conditions. WAXTS measurements were acquired by using a homemade solid‐state reactor (Figure S3, Supporting Information). The in situ solid‐state synthesis of PtCeO_2_/C catalyst was accomplished at 200 °C within a 0.5 mm quartz capillary using the commercial ceria NPs or the Ce(NO_3_)_3_ precursor. The first in situ measurement aims at replicating the synthetic conditions used to prepare the PtCeO_2_(com)/C250 sample. Nevertheless, we cannot assume that the resulting material exactly reproduces the sample prepared with lab equipment and measured ex situ because i) of the significant different volume of precursor materials, ii) the reaction is occurring in the confined space of the glass capillary, iii) temperature control with the air gun may significantly differ from that of the oven used in the synthesis of PtCeO_2_(com)/C250 sample. Therefore, such non‐negligible differences in the synthetic procedures may produce some differences in the properties of the resulting materials, e.g., particle size or size distribution. Here, more informative insights into the distinct kinetics of nucleation and growth of Pt nanoparticles through various synthetic pathways can be achieved by conducting different in situ experiments with the same experimental setup ensuring comparability of results. Therefore, the in situ experiment reproducing the reaction procedure of the PtCeO_2_(com)/C250 sample is used as a term of comparison for the second in situ experiment, monitoring the nucleation and growth of Pt NPs in the PtCeO_2_/C one‐pot synthesis. The comparison between the two in situ experiments are reported in **Figure** **3**a,e. Since the in situ experiments were performed with an operational wavelength of 0.563190 Å, the DSE analysis was performed only in the wide‐angle region. The first WAXTS measurement records the first 2 min of reaction since heating, then WAXTS measurements were acquired with a time‐step of ≈2 min until no significant changes in the X‐ray scattering pattern were observed. Preliminary analysis of the first in situ experiment (PtCeO_2_/C catalyst using commercial ceria) shows that the refinable structural/microstructural parameters (size, size dispersion, site occupancy factors, thermal atomic displacement) for ceria NPs do not show neither significant nor systematic variations with the progression of reaction, with respect to the results obtained on the ex situ analysis performed on the separate measurement of ceria NPs, providing evidence of ceria NPs stability during the growth of Pt NPs. Thus, the results previously obtained from ceria NPs (reported in Table S1, Supporting Information) were promptly transferred to the analysis of the in situ experiment reproducing the PtCeO_2_/C catalyst and were fixed during successive refinements. The only refined parameter for ceria NPs is its relative mass fraction that results to be constant at ≈15% among subsequent measurements. The average size and size dispersion of Pt NPs were refined since the constrains from the SAXS analysis were not available. Pt sub‐nanometric clusters were added as a model component and their size was refined; the WAXTS pattern of Vulcan XC72 was added as a model component and scaled. The average (number‐based) size of Pt NPs ranges from 1.9 to 2.2 nm from the first to the last measurement and size dispersion of ≈0.6 nm, showing a subtle size increase with time (Figure 3b,c). Much more evident is the increase of the relative mass fraction of Pt NPs (from 51% to 72%) at the expense of that of Pt clusters (from 32% to 13%) in the first 11 min since heating (Figure 3d). After reaching a plateau, the apparent decrease of the Pt NPs mass fraction at the end of the series and the increase of that of Pt cluster is more reasonably explained by the intrinsic limitation of this experimental setup rather than by any physical–chemical mechanism that justifies the reduction of the mass fraction of newly formed Pt NPs or promoting the formation of more unstable clusters of few Pt atoms. Indeed, considering that i) the capillary is spinning during the reaction (ensuring an ensemble of randomly oriented particles during WAXTS measurements); ii) the heating gun is focused on a small portion of the capillary to ensure a better control on the temperature of reaction; iii) the mass fraction of ceria NPs, preformed and added to the reaction mixture before heating, results to be constant within the set of measurements, we can more reasonably assume that, in this case, part of the sample contained in the capillary in adjoining regions with respect to the sample fraction fully heated by the gun moved within the capillary volume irradiated by X‐rays because of the spinning. This implies that unreacted or partially reacted sample (possibly recording a lower temperature with respect to that in the focusing volume of the heater) entered in the capillary volume of analysis, contributing with a sample fraction recording the slower reaction kinetics in terms of Pt NPs and Pt cluster mass fractions of the adjoining sample fraction during the last part of the experiment.

A comparable modeling approach was applied to the synthesis of PtCeO_2_/C without using preformed CeO_2_ NPs but by considering the nucleation and growth of CeO_2_ NPs from Ce(NO_3_)_3_ precursor (Figure 3e). In this case, the average size and size dispersion of ceria NPs were set as refinable parameters. Considering the presence of ceria NPs as a minor phase within a multiphase system, peculiar features in the WAXTS pattern characteristic of an anisotropic shape, or depending on the site occupancy factors, are not accessible (Figure 3f,g). Therefore, ceria NPs have been modeled with a univariate population of atomistic models of spherical NPs, with the diameter *D* and its size dispersion as refinable parameters. The average (number‐based) size of ceria NPs, ≈3.5 nm, and size dispersion, ≈1.4 nm, are almost constant with the progression of the reaction, being significantly smaller than the commercial ones. The mass fraction of ceria NPs for subsequent measurements is 6.5%. The relative mass fraction of Pt clusters (from 54% to 36%) significantly increases compared to the first in situ experiment, paralleling the mass fraction increase of Pt NPs (from 39% to 57%). Notably, the kinetics of this reaction is faster, as the plateau is reached within 7 min since heating (Figure 3h). A subtle increase in Pt NPs size was observed (Figure 3f,g), although the impact of time on the size and dispersion of Pt NPs is notably smaller than in the previous in situ experiment involving Pt NPs growing on commercial ceria NPs. In this case, the average (number‐based) size of Pt NPs, ranging from 1.7 to 1.8 nm, is smaller, whereas a slight increase in the size dispersion, ranging from 0.5 to 0.7 nm from the first to the last measurement, indicates a larger fraction of small‐sized Pt NPs and longer tails in the lognormal size distribution comprising few larger NPs (up to 7.5 nm in diameter). The total SSA calculated for Pt NPs (weighted for their number‐based distribution) over time for the two in situ experiments (Figure 3i) highlights the smaller size of Pt NPs in the PtCeO_2_/C one‐pot synthesis, resulting in a total SSA higher with respect to the PtCeO_2_/C synthesized with the commercial CeO_2_. These results show that the one‐pot synthesis of PtCeO_2_/C appears to favor a faster nucleation of Pt NPs smaller in size and to prevent further growth over time; this, coupled to the occurrence of a significantly higher amount of sub‐nanometric Pt clusters makes the one‐pot synthesis a more promising way for obtaining more active electrocatalysts.

### 2.4 Morphological and Chemical Characterization of PtCeO_2_/C Electrocatalysts

**Figure 4:**
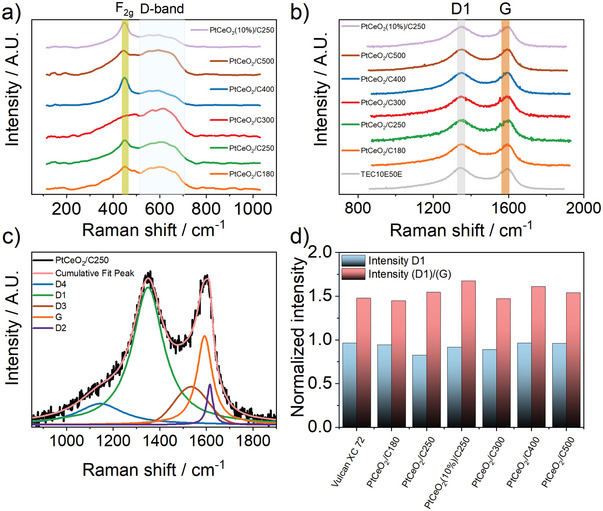
Raman response and analysis of the PtCeO_2_/C samples obtained at different temperatures and with different amounts of ceria. a) Ceria Raman bands. b) Carbon Raman bands. c) Carbon bands deconvolution (Lorentz shape for the bands D1, D2, D4, and G, Gaussian shape for the D3) of the PtCeO_2_/C180 sample. d) Comparison of the normalized intensity at 1600 cm^−1^ for the D1 bands and D1/G intensity band ratio of the carbon support in the different samples.

Raman spectroscopy was utilized to gain insights into both the carbon and ceria. Concerning this last aspect, Raman spectra of CeO_2_ depend on its structural state (single crystal, nanocrystallized, nanostructured, reduced state), and eventually defects (oxygen deficiencies) or doping could change the vibration bands bringing new shape to the canonical spectra. CeO_2_ crystallizes in the cubic fluorite structure corresponding to the space group 225/O_5_^h^ ($Fm\bar{3}m$). Group theory predicts one triply degenerate Raman active optical phonon (F_2g_ symmetry) and one infrared‐active optical phonon (F_1u_ symmetry), which presents either transverse optical and longitudinal optical character (with different wavenumbers) depending on the relative propagation/polarization directions of the mode.^[^ ^46^ ^]^ The F_2g_ band, at room temperature, is located at 466 cm^−1^ and is associated with both the Ce─O and O─O vibration bands. However, it is commonly attributed to a generic symmetrical stretching vibration of Ce–O8 units.^[^ ^47^ ^]^ At the nanoscale, this band undergoes widening, accompanied by asymmetry and a red shift.^[^ ^48^ ^]^ The presence of defects is one of the factors contributing to this broadening ≈550–600 cm^−1^ and is called “the D‐band” (D as defects). Such defects could be Frenkel‐type oxygen defects, which were shown to be present in nanocrystalline CeO_2_: an oxygen atom is then displaced from its lattice position to an interstitial site.^[^ ^49^ ^]^ **Figure** **4**a reports the Raman bands of ceria in PtCeO_2_/C samples, which are in fair agreement with examples spectra reported in the literature for different types of ceria nanostructures.^[^ ^50^, ^51^, ^52^ ^]^ The observed spectra not only reveal the existence of a broad D band but also indicate a red shift in the F_2g_ peak. This shift is attributed to a lattice distortion caused by the substantial presence of Ce^3+^ ions and, consequently, the occurrence of oxygen vacancies in the material.^[^ ^50^ ^]^ This distinction is prominent to the extent that Ce^3+^ becomes the exclusive species in the PtCeO_2_/C250 samples, in agreement with the X‐ray photoelectron spectroscopy (XPS) data discussed below. On the other hand, the presence of a large D band could be also due to the overlap of the oxygen vacancies and the contribution of the two asymmetric and symmetric stretching vibrations of bridging Pt–O–Ce groups at the Pt/CeO_2_ interface. More in detail, such bands are located ≈550 and 690 cm^−1^.^[^ ^53^ ^]^ The presence of the D‐band strongly depends on the synthesis method. In fact, comparing the Raman spectra of PtCeO_2_/C250 with PtCeO_2_(com)/C250 (Figure S4, Supporting Information), both normalized to the F₂_g_ peak intensity of ceria, reveals a lower degree of defective sites in the latter sample. This finding reinforces the beneficial effect of the one‐pot solid‐state synthesis over the simple addition of ceria nanoparticles to the carbon support. The defectivity of the one‐pot ceria not only enhances catalytic activity but also improves stability by providing an additional source of electrons.

The insights gained from these last aspects suggest that the ceria NPs, synthesized through solid‐state one‐pot methods, exhibit an enhanced metal–support interaction.^[^ ^54^ ^]^ Figure 4b displays the Raman response for the carbon supports in the Pt catalysts synthesized in different experimental conditions along with the spectrum of the sole Vulcan XC72, while Figure 4c shows a deconvolution of a spectrum, provided as an example. The carbon spectrum response was deconvoluted with four Lorentzian‐shaped bands: G, D1, D2, and D4, and with a further Gaussian‐shaped band D3, as suggested by Sadezky et al.^[^ ^55^ ^]^ The most intense D1 band appears at ≈1360 cm^−1^ and corresponds to a graphitic lattice vibration mode with A_1g_ symmetry. Another first‐order band contributing to structural disorder is the D2 band at ≈1620 cm^−1^, which can be observed as a shoulder on the G band. Similar to the G band, the D2 band corresponds to a graphitic lattice mode with E_2g_ symmetry. The D3 band is attributed to the component in between the two ends of the forceps and originates from the amorphous carbon fraction, which includes organic molecules, fragments, or functional groups.^[^ ^56^ ^]^ Finally, the shoulder at ≈1200 cm^−1^, designated as D4, could be attributed to sp^2^–sp^3^ bonds or C─C and C═C stretching vibrations of polyene‐like structures. The intensity of the D1 band, along with the D1/G band ratio, exhibits only statistical variation across samples both before and after the thermal treatment for Pt/CeO_2_ deposition, as evident in Figure 4d, and further supported by the data reported in Table S2 of the Supporting Information. This confirms the unchanged condition of the carbon support following the deposition of Pt/CeO_2_.

**Figure 5:**
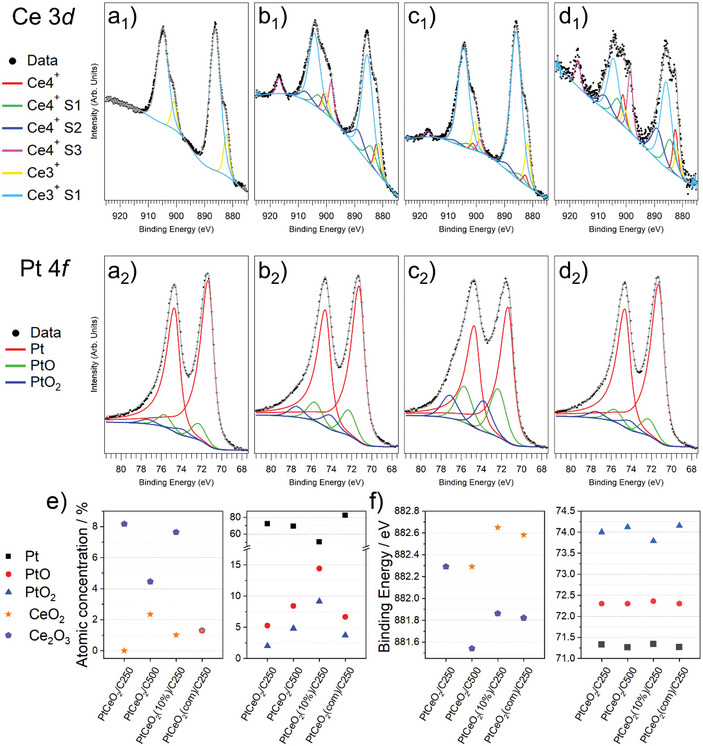
a–d) X‐ray photoelectron spectra for Ce 3d and Pt 4f core levels. CeO_2_ (a_1_–d_1_) and Pt (a_2_–d_2_) XPS signal of (a_1_,a_2_) PtCeO_2_/C250, (b_1_,b_2_) PtCeO_2_/C500, (c_1_,c_2_) PtCeO_2_(10%)/C250, and (d_1_,d_2_) PtCeO_2_(com)/C250. e) Atomic concentration of the Pt and CeO_2_ species for the samples obtained at 250 and 500 °C and with different ceria types. f) Binding energy of the Pt and CeO_2_ components for the samples obtained at 250 and 500 °C and with different ceria types.

Figure **5**a_1_–d_1_,a_2_–d_2_ shows the XPS spectra of Ce 3d and Pt 4f core levels, respectively. Concerning the Ce 3d line shape, the major difference appears in the spectrum of PtCeO_2_/C250, where the characteristic satellite due to the Ce^4+^ 3d^1/2^ emission at 917 eV binding energy (BE) is not observed (Figure 5a1). This indicates that only the Ce^3+^ oxidation state is present in the examined sample (Figure 5e), corresponding to the Ce_2_O_3_ species, or that Ce^3+^ significantly outweighs Ce^4+^ cations, associated with CeO_2_. This finding is in accordance with the presence of a large number of defects as observed by the Raman analysis (Figure 4a). In the other samples (Figure 5b1–d1), the line shape was fitted employing both Ce^3+^ and Ce^4+^ components (see Figure 5e,f for the relative intensity and BE behavior). The Pt 4f peak is instead always showing the presence of the metallic as well as both PtO and PtO_2_ components (see Figure 5e for the relative intensity and BE behavior). Note that the total concentration of oxidized Pt (PtO and PtO_2_) increases by 43% while the concentration of oxidized Ce is reduced by a factor of 3 in the PtCeO_2_(com)/C250 as compared to the PtCeO_2_/C250. This could be attributed to the larger NP size and the lower cluster density observed from the SAXS and WAXTS analysis.

The appearance of the Ce^4+^ components in the PtCeO_2_/C500 sample is accompanied by a 50% reduction of the Ce^3+^ concentration, by the BE decrease of the Ce^3+^ with respect to the PtCeO_2_/C250 case, and by an almost 50% increase of the concentration of PtO and PtO_2_. This suggests that the increase of the annealing temperature favors an oxygen transfer from the Ce_2_O_3_ to Pt phases. On the other hand, maintaining 250 °C as the annealing temperature and varying the initial CeO_2_ concentration, in the preparation procedure, result in a much larger concentration of oxidized Pt corresponding to a low atomic percentage of Ce^4+^ (1%). When employing a commercial CeO_2_ powder, the oxidation effect is instead much lower since metallic Pt dominates and both Ce^3+^ and Ce^4+^ concentrations are just around 1%. For completeness, the atomic concentration of oxygen is shown in Figure S5 of the Supporting Information. The atomic concentrations displayed in Figure 5e provide Pt/Ce ratios of 9.7, 8.6, and 35.9 for the PtCeO_2_/C250, PtCeO_2_(10%)/C250, and PtCeO_2_(com)/C250 samples, respectively, in good agreement with the results obtained from the EDX analysis (**Table** **2**).

**Table 2:** Weight (Wt) and the atomic (At) Pt/Ce ratios obtained from EDX map analysis (in the first line, the values in parentheses are the theoretical values from the mixing of the reagents).

	PtCeO_2_/C250	PtCeO_2_(10%)/C250	PtCeO_2_(com)/C250
Wt. Pt/Ce ratio	7.28 (5.00)	3.43 (2.50)	13.35 (5)
St. Dev. (s)	0.84	1.05	8.74
At. Pt/Ce ratio	10.14	4.78	27.89
St. Dev. (s)	1.17	1.47	11.48

**Figure 6:**
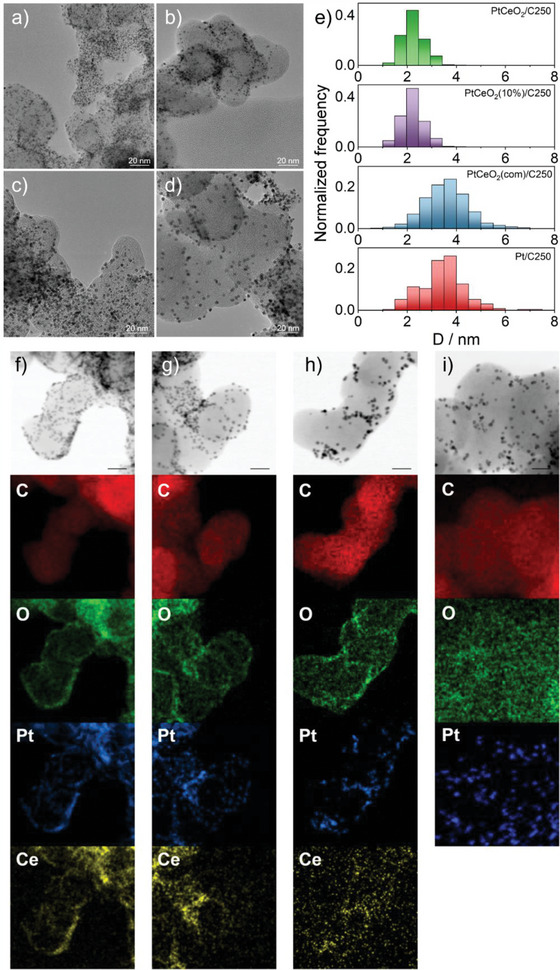
HR‐TEM images of a) PtCeO_2_/C250, b) PtCeO_2_(10%)/C250, c) PtCeO_2_(com)/C250, and d) Pt/C250. e) Particle dimensions distribution is reported in all four samples. f–i) STEM images and elemental maps of (f) PtCeO_2_/C250, (g) PtCeO_2_(10%)/C250, (h) PtCeO_2_(com)/C250, and (i) Pt/C250. From top to bottom: stem bright field image, C map, O map, Pt map, and Ce map (where present). All the images are relative to 250 °C samples. Scale bars refer to 20 nm.

The morphological characterization was accomplished by high‐resolution transmission electron microscopy (HR‐TEM). Transmission electron microscopy (TEM) images display spherical NPs with dimensions that vary depending on the different synthetic routes and the presence of Ce(NO_3_)_3_ in the reaction batch (**Figure** **6**). Crystallographic data from the Fourier transform of the HR‐TEM images, evidence only 111 and 200 reflections of Pt NPs. Indeed, the analysis of particle dimension and dispersion shows an average Pt particle diameter of ≈1.8–2 nm in samples prepared using both the Pt and Ce precursors at 250 °C (Figure 6e). The NP average diameter increases (≈3.5–4 nm) when only the Pt precursor is used, and the Ce element is introduced directly as preformed CeO_2_ NPs (Figure 6e). This implies that cerium nitrate facilitates the nucleation of platinum particles while concurrently inhibiting their growth as pointed out also by the WAXTS‐DSE analysis. This results in higher Pt atom efficiency, as smaller Pt NPs generated in the presence of cerium nitrate provide a greater electroactive exposed surface compared to larger ones obtained using only the Pt precursor. To accurately characterize the influence of temperature on PtCeO_2_/C synthesis, a comprehensive analysis of NPs size distribution was conducted for each sample as a function of the temperature synthesis. Figure S6a–e of the Supporting Information showcases representative TEM images for the samples prepared in the temperature range of 180–500 °C. The literature attests that manipulating the ramp rate can effectively control NPs size, with the smallest size achieved at the highest ramp rate.^[^ ^57^ ^]^ In the present work, an alternative methodology was employed maintaining a consistent ramp rate for all the samples while changing the synthesis temperature. This investigation not only validates the impact of the ceria precursor on NPs growth, constraining their dimensions but also illustrates that the resulting size distribution nicely scales with temperature (Figure S6f, Supporting Information). In other words, smaller NPs with a narrower size distribution can be obtained at lower temperatures compared to higher ones. This observation underscores the precise size modulation attainable through this synthesis technique.

A difference between the two resulting samples is noticeable also by observing the EDX elemental maps (Figure 6f–i), particularly about the distribution of CeO_2_, which is of significant relevance. Both the sample series produced by a one‐pot synthesis with a dual‐precursor showed a strict correlation between Pt and Ce. The EDX map indicates that Ce appears to be present in the NPs in smaller concentrations than Pt, which aligns with the synthesis procedure. Conversely, in the sample where commercial CeO_2_ is added, the simultaneous presence of both elements is not guaranteed. This was also remarked by EDX quantitative analysis of the two elements in the NPs. For each sample, ten different particle‐rich spots on the EDX maps were randomly selected and integrated, to extract mean weight (Wt) and the atomic (At) Pt/Ce ratios (Table 2). All the samples showed higher Pt/Ce values compared to those expected from the precursor concentrations, which could imply a loss of ceria during the synthetic procedure. Interestingly, PtCeO_2_/C250 and PtCeO_2_(10%)/C250 samples show low standard deviation on the Pt/Ce ratio, unlike the PtCeO_2_(com)/C250 sample, where preformed CeO_2_ was employed. These differing behaviors indicate that when Pt and Ce precursors are employed simultaneously for the nucleation and growth of both Pt and CeO_2_ NPs, a high degree of homogeneity and dispersion is achieved. Conversely, the addition of preformed CeO_2_ results in much less dispersion of CeO_2_ and lower homogeneity of the sample. This behavior can be attributed to the higher defectivity of ceria in the one‐pot sample, as demonstrated by the XPS and Raman analyses in Figures 5 and S4 (Supporting Information). These defects serve as nucleation sites for Pt while simultaneously hindering the agglomeration of Pt NPs. Furthermore, the simultaneous presence of Pt and Ce precursors in the one‐pot synthesis may also explain the absence of Ce^4+^ states, the decrease of PtO and PtO_2_ concentration, and finally the increase of the oxidized Ce concentration in the sample containing the commercial CeO_2_, as observed by XPS. This underscores the advantage of a one‐pot synthesis of Pt/CeO_2_ over the utilization of preformed CeO_2_.

### 2.5 Electrochemical Characterizations

#### 2.5.1 Conventional Activity Characterization in a Three‐Electrode Electrochemical Cell

**Figure 7:**
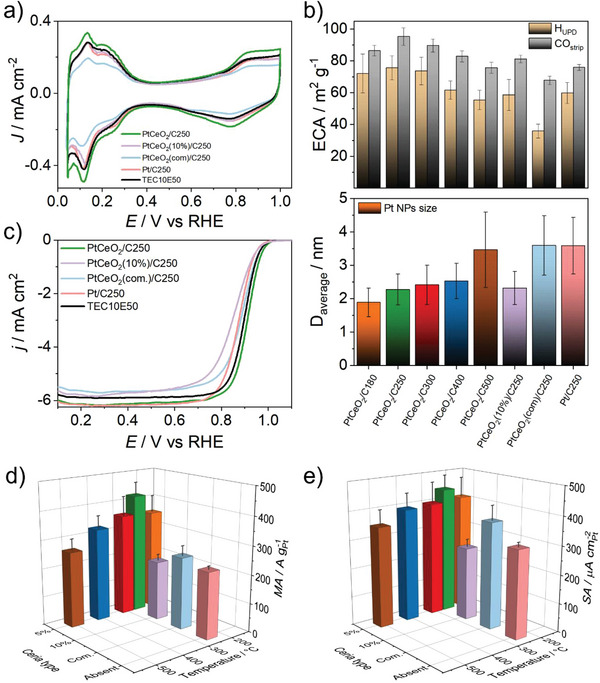
Electrochemical results in conventional three‐electrode cell configuration at 25 °C. a) CV in Ar atmosphere at 20 mV s^−1^ for the samples synthesized with different types of CeO_2_ or without the CeO_2_ at 250 °C and comparison with the standard. b) ECA collected from the H_UPD_ (gold) and from the CO_strip_ (silver) methods, aligned with the average of the diameter of the NPs in each sample, potential sweep rate of 20 mV s^−1^. c) LSV in O_2_ saturated electrolyte at 20 mV s^−1^ and 1600 rpm for the samples synthesized with different types of CeO_2_ or without CeO_2_ at 250 °C and comparison with the commercial standard (TEC10E50E). d) MA and e) SA for all the samples synthesized in this work.

The electrochemical characterizations highlighted that the activity is significantly influenced by the type of synthesis employed. After activation in an Ar atmosphere, each sample exhibited the characteristic butterfly shape of the Pt catalyst, linked to the hydrogen adsorption/desorption in the left part of the cyclic voltammetry (CV) and of OH adsorption/desorption on the right part, being the capacitive region observed in the middle (**Figure** **7**a; Figure S7a, Supporting Information). The electrochemical area (ECA) of the Pt NPs was evaluated by both hydrogen and CO stripping (a comprehensive panel of CO stripping measurements for each sample is shown in Figure S8 and data are resumed in Table S3, Supporting Information), revealing a distinctive bell‐shaped trend when correlated with the synthesis temperature. As illustrated in Figure 7b, the surface area of Pt NPs increases as the temperature rises to 250 °C by then decreasing at higher temperature, consistent with an inverse relationship of Pt size, as confirmed by the TEM analysis. A more comprehensive behavior emerges when preformed ceria or a different content of Ce precursor is used, as depicted in Figure 7a. The constraining effect obtained during the synthesis by using a ceria precursor instead of commercial ceria, influences the size and, consequently, ECA. For instance, comparing the PtCeO_2_/C250 sample with PtCeO_2_(com)/C250 and Pt/C250, when preformed commercial CeO_2_ NPs are employed, Pt NPs grow as if ceria is not present. Indeed, the ECA value from CO stripping of PtCeO_2_(com)/C250 is similar to the active surface area of Pt/C250 and the PtCeO_2_/C500 (respectively light blue, pink, and brown bar in Figure 7b), indicating notably similar dimensions. An additional comment can be made about the sample obtained at 250 °C but with a higher concentration of CeO_2_ (10%). Despite the similar size distribution of Pt NPs observed by TEM characterization, obtained under the same conditions but with 5% CeO_2_, the ECA of PtCeO_2_(10)/C250 was lower. This suggests a potential enveloping effect rather than a supporting role of CeO_2_ when present in excessive amounts thus potentially negatively impacting the active sites crucial for catalysis.

**Figure 8:**
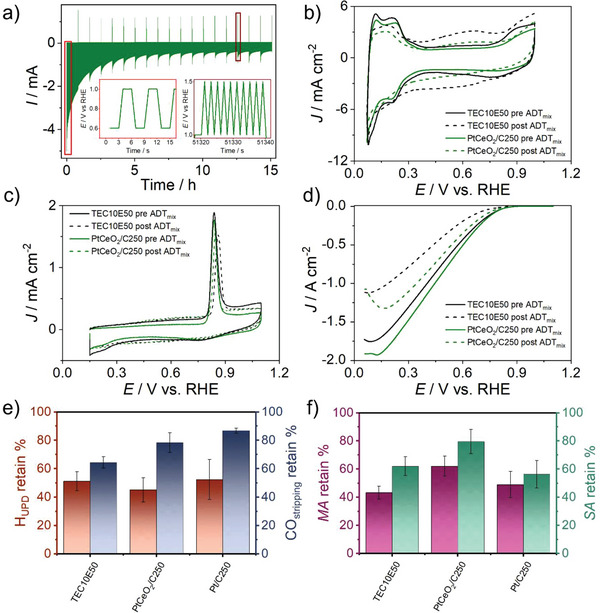
a) Stability comparison of GDE results at ambient temperature using the mixed ADT protocol. The comparison highlights the developed current under the applied potential conditions indicated in the red and wine‐colored boxes (500 potential cycles of the load‐cycling protocol followed by 10 potential cycles of the startup/shutdown protocol, repeated overall 18 times). b) CV at 500 mV s^−1^ in argon before and after the stress test between the standard TEC10E50E and the PtCeO_2_/C250. c) CO stripping first cycle before and after the stress test between the standard TEC10E50E and the PtCeO_2_/C250 collected at 50 mV s^−1^. d) LSV in O_2_ in anodic direction at 100 mV s^−1^ for the standard TEC10E50E and the PtCeO_2_/C250. e) ECA retain from CV in Ar and CO stripping measures for the standard TEC10E50E and the PtCeO_2_/C250 and the Pt/C250. f) MA and SA retain for the standard TEC10E50E and the PtCeO_2_/C250 and the Pt/C250.

The activity of every sample was assessed through the linear sweep voltammetry (LSV) at rotating disk electrode (RDE) in O_2_ saturated 0.1 m HClO_4_ electrolyte, and the activity trend follows that of the ECA (**Figure** **8**d,e). The activity trend against the synthesis temperature, derived from the LSV in Figure S7b of the Supporting Information, follows the same bell‐shaped pattern observed for the ECA. Notably, PtCeO_2_/C250 occupies the highest activity point values for both MA and SA plots (Figure 7d,e). A bell‐shaped trend is also documented in the literature, but as a function of Pt NPs size.^[^ ^58^ ^]^ It is worth stressing that the ORR activity performance of PtCeO_2_/C250 easily surpasses that of the catalysts prepared without CeO_2_, with preformed commercial CeO_2_ or with a higher percentage (10%) (Figure 7d,e). This showcases the efficacy of one‐pot synthesis over multistep synthesis and indicates that the optimal amount of CeO_2_ addition is close to 5%.

The comparison of catalysts with larger Pt NPs sizes (PtCeO_2_/C500, PtCeO_2_(com)/C250, and Pt/C250) reveals differences in activity despite similar sizes (Figure 7d,e). This suggests an improvement in O_2_ transport mechanisms from CeO*_x_* to the Pt NPs, as supported by previous literature. This mechanism, reinforced by the presence of oxygen defects evidenced by Raman and XPS analyses, is well described by Vayssilov et al. as reverse spillover.^[^ ^19^ ^]^ More in detail, in a model where a stable Pt cluster is supported by a ceria NP, a purely electronic effect involving electron transfer from Pt to CeO_2_ is accompanied by the oxygen removal from the site with the lowest vacancy formation energy and, then, transferred to the Pt cluster.

Lastly, ORR performances are compared with a typical Pt standard (TEC10E50E, **Table** **3**). It is important to note that the performances of the two standards represent the average result obtained in one case with the procedure outlined in the experimental method (using a minimal amount of Nafion to avoid mitigating activity), in the other it refers to the best standard performance found in the literature.^[^ ^59^ ^]^ Regarding the values obtained with the casting procedure adopted in this work, the Pt standard has MA_0.9 V_ and SA_0.9 V_ in line with the trend against Pt size (considering that the average diameter of Pt NPs for the standard is ≈2.6 nm). Furthermore, PtCeO_2_/C250 sample exhibits performances like the standard from the literature, which was studied using the best optimized practices to maximize activity behavior.

**Table 3:** MA, kinetic current, and SA measured for each studied sample (at 0.9 V vs RHE) compared with the literature benchmark.

Samples	MA^a)^ [mA g^−1^]	*I*_k_ [mA cm^−2^]	SA^b)^ [mA cm^−2^]
PtCeO_2_/C180	(3.5 ± 0.6) × 10^5^	5.3 ± 0.9	0.41 ± 0.07
PtCeO_2_/C250	(4.2 ± 0.5) × 10^5^	6.3 ± 0.7	0.45 ± 0.05
PtCeO_2_/C300	(3.6 ± 0.7) × 10^5^	5 ± 1	0.40 ± 0.07
PtCeO_2_/C400	(3.3 ± 0.4) × 10^5^	4.9 ± 0.6	0.40 ± 0.05
PtCeO_2_/C500	(2.6 ± 0.4) × 10^5^	4.0 ± 0.6	0.35 ± 0.05
PtCeO_2_(10%)/C250	(2.1 ± 0.2) × 10^5^	3.1 ± 0.3	0.26 ± 0.03
PtCeO_2_(com)/C250	(2.5 ± 0.4) × 10^5^	3.8 ± 0.5	0.37 ± 0.05
Pt/C250	(2.3 ± 0.12) × 10^5^	3.5 ± 0.2	0.31 ± 0.02
TEC10E50E	(2.3 ± 0.21) × 10^5^	3.5 ± 0.4	0.30 ± 0.03
TEC10E50E benchmark^[^ ^59^ ^]^	(4.8 ± 0.4) × 10^5^	8.6 ± 0.8	0.48 ± 0.04

^a)^ Mass activity referred to Pt mass;

^b)^ Specific activity referred to Pt surface area.

#### 2.5.2 Gas Diffusion Electrode: Stability Characterization

The second crucial criterion evaluated is the impact of ceria on the catalyst stability. In recent years, Arenz and co‐workers proposed a GDE cell,^[^ ^60^ ^]^ wherein the catalyst is deposited on a gas diffusion layer (GDL) with a mesoporous layer (MPL) on top. This configuration, shown in Figure S9 of the Supporting Information, simulates a fuel cell cathodic compartment, offering a more realistic setting. Arenz and co‐workers explored three ADTs on a GDE cell: a load‐cycling protocol involving square wave modulation from 0.6 to 1.0 V versus reversible hydrogen electrode (RHE) (Figure S10a, Supporting Information), with 3 s holding times (which represents a mild stress condition); a startup/shutdown protocol cycling potential from 1.0 to 1.5 V versus RHE (which is likely too aggressive and impractical, as it leads to the corrosion of the carbonaceous support, causing the detachment of a substantial portion of the catalyst); and last, a mixed protocol combining 500 cycles of load‐cycling with 10 cycles of startup/shutdown (**Figure** **8**a).^[^ ^60^ ^]^

Figure S10 of the Supporting Information presents a comparison of the standard Pt catalyst and the best performing PtCeO_2_/C250 sample under the load‐cycling protocol involving square wave modulation from 0.6 to 1.0 V versus RHE, with 3 s holding times. Figure S10b–d of the Supporting Information shows the ECA retention, an example of the LSV for both the samples and the activity retention, respectively. The ECA retention result is similar for the two samples, and the MA_0.65 V_ and SA_0.65 V_ activity losses are nearly zero for PtCeO_2_/C250 whereas decrease to 80–90% for the Pt/C benchmark. These findings attest to the inadequacy of this protocol for testing the stability induced by CeO_2_. Therefore, the mixed protocol was adopted to replicate the typical startup–shutdown behavior of a fuel cell. Figure 8a illustrates this protocol, highlighting the two distinct applied voltages during the stress test (marked by the red and wine‐colored boxes) and the corresponding current response.

During shutdown, the anode compartment is typically purged with air to replace hydrogen, creating an equilibrium state at both electrodes. This approach mitigates degradation during idle periods and enhances system stability over extended downtimes. However, upon startup, when hydrogen is reintroduced into the anode, a hydrogen–air front forms and propagates through the anode compartment. This transient coexistence of gases with different equilibrium potentials at the same electrode induces a reverse current in specific regions of the fuel cell, accelerating degradation.^[^ ^61^ ^]^ The reverse current increases the cathode interfacial potential difference, leading to the formation of undesirable byproducts such as such as H_2_O_2_, hydroxyl radicals (HO^•^), hydroperoxyl radicals (OOH^•^), and carbon corrosion, ultimately compromising the cathode electrode structure.

The effects of startup/shutdown degradation, on the measured samples, are further demonstrated through CV under Ar flow. After the mixed ADT, a significant decrease in the H_UPD_ is observed. Additionally, a noticeable impact on the quinone/hydroquinone redox couple peak at 0.6 V is evident, accompanied by a shift in the Pt/C features (Figure 8b). These changes are attributed to carbon corrosion, affecting both the Vulcan carbon and the GDL carbon structure.

These findings emphasize the unreliable ECA determined after the ADT from the H_UPD_ charge, due to the effect of carbon corrosion on the CV shape and thus on the H_UPD_ region. In fact, a comparison of the ECA retention obtained from the H_UPD_ region with that from the CO stripping (Figure 8c) indicates that the CO stripping method is unaffected by corrosion contributions. Therefore, the CO stripping method was adopted for ECA determination for all the samples (Table S3, Supporting Information). Evidence of loss activity after the mixed protocol ADT comes also from the LSV under O_2_ flow, where a sensitive diminution of the ORR reduction current can be observed (Figure 8d).

To assess the impact of CeO_2_ on stability, the retention of the area measured during CO stripping reveals a value of 64% ± 4% for the standard, consistent with the literature and lower than the PtCeO_2_/C250 sample, which recorded 78% ± 6% (see Figure 8e; Table S4, Supporting Information).^[^ ^60^ ^]^ The activity mirrors the ECA behavior, passing from an MA_0.65 V_ and an SA_0.65 V_ retention of 43% ± 5% and 62% ± 7%, respectively, for the standard, to an MA_0.65 V_ and an SA_0.65 V_ retention of 62% ±7% and 79% ± 9% for the PtCeO_2_/C250 (see Figure 8f; Table S4, Supporting Information). The stability performance derived from ceria was further compared with that of the sample synthesized at 250 °C without CeO_2_ (depicted in Figure 8e,f and detailed in Table S4, Supporting Information). The sample without CeO_2_ exhibits a comparable ECA retention to PtCeO_2_/C250, but in terms of MA_0.65 V_ and SA_0.65 V_ Pt/C250 remains undoubtedly inferior (MA_0.65 V_ = 49% ± 9% and SA_0.65 V_ = 56% ± 10%). It is worth stressing that the Pt NPs of Pt/C250 are almost twice in dimension compared to PtCeO_2_/C250, as discussed in the previous paragraph. This size difference likely influences the dissolution/aggregation of NPs, as suggested by Su and co‐workers,^[^ ^62^ ^]^ who found that transitioning from 2.5 to 3.5 nm results in halved electrochemical surface area loss. Additionally, Arenz and co‐workers demonstrated a significantly higher loss in ECA for catalysts with smaller particle sizes (2–3 nm, TEC10E50E) compared to those with larger sizes (4–5 nm, TEC10E50E‐HT).^[^ ^60^ ^]^

Under these assumptions, it can be asserted that CeO_2_ NPs have a beneficial effect, enhancing stability to compete against samples with larger Pt NPs dimensions. This assumption is further supported by the fact that PtCeO_2_/C250 samples still exhibit the highest activity retained, providing additional evidence of the beneficial effect of CeO_2_ in this regard.

The comparable outcomes observed in the load cycle test for both samples, the Pt/C standard and the PtCeO_2_/C250, along with the encouraging stability demonstrated in the mixed protocol for PtCeO_2_/C250, outline the CeO_2_ effectiveness in enhancing stability, particularly in oxidative stress conditions. This is likely attributed to the capacity of the sub‐stoichiometric CeO*_x_*, whose presence in the PtCeO_2_/C250 sample has been amply demonstrated in the previous sections, to mitigate the undesired byproducts obtained in the ADT, acting as a radical scavenger conditions through reactions such as^[^ ^63^ ^]^

(2) $$Ce^{4+}+H_{2}O_{2}⇆Ce^{3+}+\mathrm{HO}O^{\cdot }+H^{+}$$

(3) $$Ce^{4+}+\;\mathrm{HO}O^{\cdot }⇆\;Ce^{3+}+O_{2}+H^{+}$$

(4) $$Ce^{3+}+H^{+}+\;OH^{\cdot }⇆\;Ce^{4+}+H_{2}O$$

(5) $$Ce^{3+}+H^{+}+\mathrm{HO}O^{\cdot }⇆\;Ce^{4+}+H_{2}O_{2}$$

where Ce^4+^ facilitates the decomposition of hydrogen peroxide and peroxyl radicals, while Ce^3+^ scavenges hydroxyl and peroxyl radicals, thereby curbing the comprehensive corrosion of both the carbon support and the Pt catalyst.

## 3 Conclusion

This paper presents a novel one‐pot synthesis of Pt and CeO_2_ NPs on carbon Vulcan XC72 and the in‐depth investigation of the effect of CeO_2_ on the catalytic activity and stability of Pt NPs versus the oxygen reduction reaction. It was finely clarified how the synthetic procedure affects the nucleation and growth of Pt NPs depending on whether CeO_2_ is included as preformed NPs in the reaction vessel, or it is allowed to nucleate and grow along with Pt NPs. Furthermore, it was observed that Pt NPs form, even at low temperatures (180 °C) under the effect of the temperature and without the presence of hydrogen as a reducing agent. This last aspect was elegantly followed by WAXTS‐DSE analysis, which suggests that in the one‐pot synthesis, Pt NPs form more rapidly, than in the presence of preformed CeO_2_, and the reason lies in the concomitant formation of more defective CeO_2_ NPs. Furthermore, Pt NPs remain smaller, ≈2 nm, and much better dispersed over the carbon support while maintaining an elevated high surface area. HR‐TEM analysis confirmed that the one‐pot synthesis yields a Pt NPs dispersion that closely matches the one of CeO_2_ NPs on the carbon support, a situation that is not achieved if CeO_2_ is included as preformed NPs.

Raman and XPS analyses revealed the presence of oxygen vacancies in the PtCeO_2_/C samples prepared through one‐pot synthesis. Furthermore, it was observed that the concentration of oxygen defects varies depending on the synthesis temperature. Specifically, at lower temperatures (250 °C), a higher number of oxygen defects and a simultaneously increased amount of Pt oxide were detected. This suggests a metal–support interaction between Pt and CeO_2_, easily exchanging electrons and oxygen, which facilitates the nucleation and growth of Pt NPs. Subsequent electrochemical characterization confirmed that PtCeO_2_/C electrocatalysts exhibit high activity toward the oxygen reduction reaction, and this activity correlates well with the size and electrochemical surface area of the Pt NPs. Furthermore, the best‐performing catalysts PtCeO_2_/C250 (MA = 423 A g_Pt_^−1^; SA = 446 µA cm^−2^) is as active as the commercial benchmark Pt/C but much more stable according to accelerated stress tests performed on GDE electrodes. In fact, the MA and SA retentions are 62% and 79%, respectively, for PtCeO_2_/C250, versus 49% and 56% for the literature benchmark, respectively.

## 4 Experimental Section

### Chemicals

Ultrapure water (resistivity > 18.2 MΩ cm) was used for acid/base dilutions, catalyst ink formulation, and the GDE cell cleaning. The following chemicals were used in ink formulation and electrolyte preparation: isopropanol (IPA, 99.7%, Alfa Aesar), Pt(acac)_2_ (Sigma‐Aldrich, > 97%), Ce(NO_3_)_3_·6 H_2_O (Sigma‐Aldrich, 99% trace metal basis), carbon black (Vulcan XC 72), CeO_2_ NPs (Particular Materials diameter 10 nm); Nafion (Sigma‐Aldrich, 5% by weight in EtOH), NaOH (VWR, >99%), HClO_4_ (Fluka, Traceselect 67–72%), and acetone (Fluka, HPLC grade) were used as received without further purification. AlphaGaz O_2_, Ar, and N_2_ were supplied by Air Liquid at the highest available purity (>99.99%). A commercial 46.5 wt% Pt/C catalyst (TEC10E50E, Tanaka kikinzoku kogyo) was used as standard. The Nafion membrane (Nafion 117, 183 mm thick, Fuel Cell Store) and two GDL, one with an MPL (Freudenberg H23C8), and one without the MPL (Toray TP120) were employed in the GDE cell measurements. Before use, the Nafion membrane was prepared and activated as follows: after cutting several circles with a diameter of 2 cm from a sheet of Nafion membrane, the membranes were treated for 30 min at 80 °C in 5 wt% H_2_O_2_, followed by rinsing with Milli‐Q water. Then, the membranes were treated for 30 min at 80 °C in Milli‐Q water followed by rinsing with Milli‐Q water. Finally, the membrane was treated for 30 min at 80 °C in 8 wt% H_2_SO_4_, again followed by rinsing with Milli‐Q water. All membranes were kept in a glass vial filled with Milli‐Q water. After every use, the membranes were rinsed in boiling water trice for 30 min.

### One‐Pot Solid‐State Synthesis

PtCeO_2_/C samples were prepared weighting in a 20 mL beaker Pt(acac)_2_, Ce(NO_3_)_3_·6 H_2_O, and carbon black in order to have an expected final catalyst weight of 125 mg with a theoretical Pt content of 25%, a CeO_2_ content of 5%, and the remaining 70% of carbon. 20 mL of acetone were added to dissolve the salts, suspending the carbon portion. The acetone was dried under agitation with a magnetic stirrer (AREX Velp) during the night, helped by a hot plate set at 80 °C. The collected dried powder was then homogenized by pressing and mixing all the precursors in a mortar and put in a quartz reactor in a vertical configuration. Once the gas connection was guaranteed, N_2_ gas was flowed at 100 mL min^−1^ (sccm) for an hour in ambient temperature to saturate the reactor with the inert gas. Next, the reactor was heated in a furnace (Carbolite, UK), with a heat ramp of 10 °C min^−1^ reaching 100 °C, and maintaining this temperature for an hour, to eliminate water traces. After that, a ramp rate of 20 °C min^−1^ was used to reach the temperature of synthesis, which was kept for 3 h. Five temperatures of synthesis were tested: 180 °C, 250 °C, 300 °C, 400 °C, and 500 °C, which gave the name to the respective samples. To get a full panel of the effect of ceria other three samples were prepared using a synthesis temperature of 250 °C: one with the absence of ceria using a 75% of theoretical Vulcan and a 25% Pt named Pt/C250; one with an amount of Ce(NO_3_)_3_·6 H_2_O calculated to obtain a 10% of theoretical CeO_2_, 25% of Pt, and the 65% of Vulcan, named as PtCeO_2_(10%)/C250; one with a 5% of commercial CeO_2_ NPs (Particular Materials diameter 10 nm) instead of the ceria precursor, named as PtCeO_2_(com)/C250.

### Synchrotron WAXTS Measurements

WAXTS measurements were performed at the X04SA‐MS beamline of the Swiss Light Source (Paul Scherrer Institut, Villigen, CH).^[^ ^64^ ^]^ The beam energy was set at 22 KeV and operational wavelength *λ* = 0.563291 Å was determined by measuring a silicon powder standard sample (NIST 640c). WAXTS data were collected in transmission mode in the 0.07–19 Å^−1^ *Q* range (where *Q* = 4πsin*θ*/*λ*, 2*θ* is the scattering angle), using a single‐photon counting silicon microstrip MYTHEN III detector, on the samples loaded in a 0.3 mm diameter glass capillary. Separate measurements on the Vulcan XC72 and the commercial CeO_2_ NPs were also performed. Separate air and empty capillary scattering measurements were collected. The transmission coefficient of the samples was experimentally determined by measuring the direct and transmitted beams, whereas that of the glass capillary was calculated from the certified composition and wall thickness of the capillary. Raw data were corrected for systematic errors and absorption effects; the extrasample contributions to the scattering pattern, i.e., the scattering from the capillary and the sample environment, were subtracted. The reduced WAXTS data containing only the sample scattering pattern were analyzed through the total scattering approach based on the Debye scattering equation. DSE‐based refinements were performed in the 2.5–19 Å^−1^ *Q* range.

### SAXS Measurements

The SAXS signal was not recorded through a dedicated SAXS instrument, but it was acquired at the X04SA‐MS beamline, maintaining the same experimental setup used during the WAXTS experiment, but changing the beam energy at 8 KeV. The operational wavelength *λ* = 1.549188 Å was determined by measuring a silicon powder standard sample (NIST 640c). This new experimental setup enables one to extend the low‐*Q* range of the X total scattering measurement, acquiring high‐quality data in the 0.024 –0.5 Å^−1^ *Q* range. The raw data correction and reduction followed the same procedure as for the WAXTS measurements.

### The DSE Method

The Debye scattering equation^[^ ^29^, ^30^ ^]^ enables the computation of the total X‐ray scattering pattern in the reciprocal space of randomly oriented NPs from the distribution of interatomic distances within atomistic models. The simulated scattering pattern is calculated as

(6) $$I\;\left(Q\right)=\sum_{j=1}^{N}f_{j}{\left(Q\right)}^{2}o_{j}+2\sum_{j>i=1}^{N}f_{j}\left(Q\right)f_{i}\left(Q\right)T_{j}\left(Q\right)T_{i}\left(Q\right)o_{j}o_{i}\frac{\sin\;\left(Qd_{ij}\right)}{\left(Qd_{ij}\right)}$$

where *Q = 4*πsin*θ*/*λ* is the magnitude of the scattering vector, *λ* is the radiation wavelength, *f_j_* is the X‐ray atomic form factor of atom *j*, *d_ij_* is the interatomic distance between atom *I* and *j*, and *N* is the number of atoms in the NPs. *T_i_* and *o_i_* parameters refer to the thermal atomic displacement and site occupancy, respectively. The first summation accounts for the contribution of the zero distances of each atom from itself, whereas the second summation accounts for the nonzero distances between pairs of distinct atoms. The DSE modeling was carried through a bottom‐up approach within the DebUsSy Suite.^[^ ^65^ ^]^ Populations of atomistic models of nanocrystals at increasing size and desired shape were generated according to one (*D*) or two (*D*_ab_, *L*_c_) growth directions in the case of isotropic or anisotropic shapes, respectively. Gaussian sampled interatomic distances and pseudomultiplicities were encoded in databases used to simulate the scattering pattern through the Debye equation.^[^ ^66^ ^]^ The calculated scattering pattern was generated by the weighted sum of the patterns from each atomistic model according to their number fraction of lognormal size distribution. The calculated pattern was compared to the experimental one and the difference between them was minimized using a simplex algorithm by refining a number of adjustable structural and microstructural parameters in the model. The number‐based average size and size dispersion of a lognormal size distribution function along one or two independent growth directions (in the case of isotropic or anisotropic NPs, respectively) were optimized. The isotropic atomic thermal displacement (Debye–Waller *B* factor) was also refined. The goodness of fit (GoF, the square root of reduced *χ* ^2^) is the statistical descriptor used to evaluate the agreement between calculated and experimental patterns. The WAXTS pattern of the Vulcan‐XC72 was separately measured and added as an additional phase in the DSE analysis and used as background, accounting for the X‐ray scattering of the carbon support within the analyzed samples.

### The Atomic Pair Distribution Function Method

The PDF represents another approach to the analysis of X‐ray total scattering data, providing the probability of finding pairs of atoms separated by a distance *r* in real space.^[^ ^42^ ^]^ This method is particularly suited to investigate the short‐range order of amorphous and nanostructured materials, extracting structural information without any assumption on periodicity. Here the difference PDF was applied to the amorphous‐like trace that has been added as a background (together with the WAXTS pattern of the Vulcan‐XC72) during the DSE refinement, compensating the difference between the calculated and experimental patterns and modeled through a polynomial function. The analyzed trace was calculated in the reciprocal space as the difference between the total background and the WAXTS signal of the carbon support. The total scattering structure function *S*(*Q*) was calculated using PDFgetX3,^[^ ^67^ ^]^ then the atomic PDF was obtained through the sine Fourier transform of the reduced total scattering structure function *F*(*Q*) = *Q*[*S*(*Q*)‐1] as

(7) $$G\left(r\right)=\frac{2}{\pi }\int_{Q_{\min}}^{Q_{\max}}Q\left[S\left(Q\right)-1\right]\sin\left(Q_{r}\right)dQ$$

where *Q* = 4πsin*θ*/*λ* is the magnitude of the scattering vector. *Q*_min_ = 1.6 Å^−1^ was set equal to that used for the DSE analysis and *Q*_max_ = 18.0 Å^−1^ was determined by the experimental setup. Despite the experimental setup is not specifically tailored for PDF data acquisition (instead it is optimized for the DSE approach) and the *Q*_max_ value is slightly lower than that obtained from proper PDF experiments, the main structural correlations within the material can be reliably extracted.^[^ ^43^, ^68^ ^]^

### One‐Pot In Situ Reactor Solid‐State Synthesis

To monitor the nucleation and the growth of Pt and CeO_2_ during the synthesis, an in situ XRD (synchrotron light) characterization was performed. This was achieved by exploiting the acquisition time and the resolution of the WAXTS measurements conducted through the X04SA‐MS beamline. The process was carried out thanks to the home‐made reactor, as illustrated in Figure S3 of the Supporting Information, where the rotating capillary holder is connected to a rotor (Metal gear motor 6 Vdc – 130 rpm), which rotates the capillary to allow a well irradiation of the powder inside the capillary. A hot gun, set to 200 °C, and focalized to the center of the capillary, in concomitance with the point where X‐ray impinges on the capillary, allowed to heat the sample promoting the degradation of the precursor and the growth of the NPs. A thermocouple and a thermal camera (FLIR AX8) allowed the control of the temperature before and during the measure. With this specific setup, a comparative analysis of two distinct PtCeO_2_/C synthesis methods was conducted. The primary objective was to investigate the variances in nucleation and NPs growth as a function of the different CeO_2_ additions. This study aimed to simulate the synthesis of PtCeO_2_(com)/C alongside the synthesis of PtCeO_2_/C in a one‐pot process, where both the Pt and CeO_2_ precursors are combined within the same reactor.

### Electrochemical Analysis in Rotating Disk Electrode

PtCeO_2_/C catalysts were electrochemically characterized in a three‐electrode configuration cell, using a Potentiostat/Galvanostat Biologic SP‐300. The catalysts were tested in a 0.1 m HClO_4_ solution at 25 °C. A Glassy Carbon GC electrode (5 mm diameter, geometric surface area 0.196 cm^2^) was used as a working electrode. A platinum electrode was used as the counter electrode and an RHE was used as a reference electrode.^[^ ^69^ ^]^ The catalysts were electrochemically characterized as films, drop casted on the working electrode. The catalyst ink was formulated for a single drop cast of ≈12 µL, obtaining a final platinum loading on the electrode of 15 µg cm^−2^. The ink composition was prepared as follows: 2.2 mg of nanocomposite sample was added to 2 mL of H_2_O Milli‐Q, 0.2 mL of isopropanol, and a 1/1 rate in weight of Nafion solution, and the resulting suspension was ultrasonicated for 30 min at 25 °C. The catalysts were then carefully drop casted onto a clean GC surface and allowed to dry in rotation using a rate of 400 rpm at room temperature. The electrolyte was purged with Ar before each measurement, while for the ORR test, the electrolyte was bubbled with high‐purity O_2_ gas for at least 30 min to ensure O_2_ saturation. The catalysts were activated by performing 100 voltammetry cycles at 200 mV s^−1^ between 0.02 and 1.2 V versus RHE or until stable cyclic voltammograms were obtained. Catalytic activity versus ORR was evaluated by recording linear sweep voltammograms at a scan rate of 20 mV s^−1^ from 0.05 to 1.05 V versus RHE in the electrolyte saturated with O_2_ using the RDE technique.^[^ ^70^ ^]^

The electrochemical platinum surface area (ECA) was calculated from both hydrogen (H_upd_) and carbon monoxide (CO_strip_) desorption regions, using, respectively, a charge of 210 and 410 µC cm^−2^. The ECA for the H_upd_ was evaluated by recording several CVs at 20 mV s^−1^ in argon‐saturated solution. On the other hand, the ECA from the CO_strip_ is measured at 20 mV s^−1^. The electrochemical surface area was calculated using the following formula

(8) $$\mathrm{ECA}\;\left(cm_{\mathrm{Pt}}^{2}/g_{\mathrm{Pt}}\right)=\frac{\mathrm{Charge}\;}{0.21\;*\;\mathrm{electrode}\;\mathrm{loading}}$$

The kinetic current density *j_k_* was determined using a rotating electrode rotated at 1600 rpm. The current density was taken at 0.9 V versus RHE and corrected by mass transfer, according to

(9) $$j_{k}=\frac{|j_{\lim}\left|\cdot \right|j_{0.9\;V\;\mathrm{vs}\;\mathrm{RHE}}|}{|j_{\lim}\left|-\right|j_{0.9\;V\;\mathrm{vs}\;\;\mathrm{RHE}}|}$$

The catalysts’ activity was evaluated considering the mass activity and the specific activity obtained through the following equation

(10) $$\mathrm{MA}=\frac{j_{k}}{m_{\mathrm{Pt}}}\;$$

(11) $$\mathrm{SA}=\frac{j_{k}}{\mathrm{EC}A_{{\mathrm{CO}}_{\mathrm{strip}}}*\;m_{\mathrm{Pt}}}\;$$

where $\mathrm{EC}A_{{\mathrm{CO}}_{\mathrm{strip}}}$ is the ECA obtained from the CO stripping measurements, instead the *m*_Pt_ is the quantity of Pt on the electrode used, corrected with the mass revealed through inductively coupled plasma mass spectrometry analysis (ICP‐MS). The ORR kinetic current was evaluated through RDE polarization curves, subtracting background surface oxidation and capacitive processes using the CV recorded in the Ar‐saturated solution obtained with the same experimental parameters (i.e., scan speed, rotation rate, potential window). All electrochemical measurement was recorded with the *iR* drop compensation.^[^ ^71^, ^72^ ^]^

### Electrochemical Analysis and Stress test in Gas Diffusion Electrode Cell

The durability and stability of the PtCeO_2_/C catalyst were verified through an ADT on a gas diffusion electrode cell designed by Arenz and co‐workers^[^ ^31^ ^]^ (Figure S9, Supporting Information), where Pt and RHE electrodes were used respectively as counter and reference electrodes. For the deposition of the catalyst on the GDL, a 10 mL water/IPA ink, with 25% of IPA and 10 µL per mg of Nafion (5% wt) was prepared and sonicated for 30 min. The amount of catalyst per ink was calculated to obtain a loading of ≈10 µg cm^−2^ deposit via spray coating on a square GDL of 16 cm^2^. The catalyst ink was sprayed onto the GDL (MPL‐coated side) using a spray coater (ND‐SP Ultrasonic Spray Coater, Nadetech Innovations) with a flow rate of 10 mL h^−1^ and an ultrasonic spray nozzle with a power of 2.9 W, a frequency of 90 400 Hz, and pressure of 0.05 bar, and the GDL was heated on a heating plate (140 °C). From the deposited GDL a circular piece of 3 mm in diameter was punched. This piece was positioned in a new pristine GDL presenting a concentric hole of the same diameter.

This setup facilitated precise control of the deposited area in a reproducible manner, ensuring consistent use of the same amount of catalyst for sequential tests. Under such assembled GDL, a carbon paper served as the base, with a Nafion membrane positioned between the catalyst layer and the liquid electrolyte. The upper cell body is pressed to the lower cell body by a metal ring and a clamp. A gas humidifier (gas bubbler) is connected to the reactant gas. Notably, no liquid electrolyte came into direct contact with the catalyst but only diffused through the membrane.

For the initial cleaning process, the Teflon upper part was immersed in a mixed acid solution (H_2_SO_4_:HNO_3_ = 1:1, v/v) overnight. Subsequently, it underwent thorough rinsing with ultrapure water and was boiled in ultrapure water twice. In between measurements, the Teflon upper part underwent additional boiling in ultrapure water twice, ensuring proper cleaning and maintenance of experimental conditions.

The catalysts were tested in a 4 m HClO_4_ (to limit the ohmic contribution) solution at ambient temperature. A platinum electrode was used as a counter electrode and an RHE was used as a reference electrode.

Before conducting measurements, the catalyst underwent a two‐step activation process. Initially, potential cycles ranging between 0.06 and 1.10 V versus RHE at a scan rate of 500 mV s^−1^ were applied in an O_2_ atmosphere until a stable CV was achieved, typically requiring ≈50 cycles. Subsequently, an additional 50 cycles were performed in an Ar atmosphere for further stabilization. The initial activation in oxygen aimed to enhance Nafion membrane adhesion to the catalyst by facilitating water production from O_2_ reduction. This prevented inert gas bubbles from interposing between the catalyst and the membrane, thereby avoiding unwanted ohmic contributions. Following stabilization in Ar, the resistance from the WE to the reference was measured using impedance spectroscopy and carefully compensated before each performance measurement.

CO desorption features were collected by flowing CO directly into the backside of the cell, avoiding the humidification path to prevent potential contamination of CO inside the humidifier. A potential of 0.05 V versus RHE was applied for 600 s, with the first 90 s saturating all active sites with CO. Subsequently, the CO flow was stopped and replaced with a humidified Ar flow for the remaining 510 s to eliminate any traces of CO that could compromise the measurement. Next, 10 cycles at 500 mV s^−1^ were applied, starting from 0.4 V versus RHE in a range from 0.15 V versus RHE to 1.1 V versus RHE. The CO stripping measure was the first performance measurement to avoid potential changes due to active site poisoning in the retention calculation. Following the CO stripping measure, a new activation with 50 potential cycles ranging between 0.06 and 1.10 V versus RHE at a scan rate of 500 mV s^−1^ was applied to prepare the catalyst for the H_upd_ measurement. For H_upd_, 3 cycles at 500 mV s^−1^ were applied in a range between 0.07 V versus RHE and 1 V versus RHE.

Finally, the gas flow was shifted from Ar to O_2_, and a series of cycles from 0.05 V versus RHE to 1.1 V versus RHE at 100 mV s^−1^ were applied until a stable feature was obtained.

Two accelerated degradation protocols were tested.^[^ ^60^ ^]^

1. A protocol simulating load cycles, where the electrode potential was modulated with a square wave and stepped between 0.6 and 1.0 V versus RHE with a holding time of 3 s at each voltage for a total of 9000 potential cycles.
2. A mixed protocol combining 500 potential cycles of the load‐cycling protocol followed by 10 potential cycles of the startup/shutdown protocol, where the electrode potential was cycled with a scan rate of 500 mV s^−1^ between 1.0 and 1.5 V versus RHE, repeated overall 18 times.

After the ADT, performance measurements were collected in reverse order, starting from the activity measure in O_2_, followed by measurements in Ar conditions, starting with H_upd_ and concluding with the CO stripping area.

### X‐Ray Photoemission Spectroscopy

The system composition was evaluated by XPS using an Mg X‐ray source (incident photon energy 1253.6 eV) and a Phoibos 100 SPECS analyzer at 20 eV pass energy, providing an overall resolution of 0.9 eV. The BE was calibrated referencing the C1s graphitic component at 284.4 eV. The spectral deconvolution was performed employing CASA Xps software, adopting a Tougard background and the GL50 line shape for all the components (Ce 3d, PtO, PtO_2_, O, and C), except for the metallic Pt components, for which an LA line shape was used. For Ce^4+^, the Ce 3d components (main peak and satellites) BE, width (FWHM), relative BE separation, and intensity ratio of the satellites were determined by acquiring XPS spectra of pure CeO_2_ powders on the same experimental setup and comparing the data with the literature.^[^ ^73^ ^]^ To avoid modifications of the powder by the X‐ray beam, a single scan was performed. Ce^3+^ components were estimated in the same way as acquiring the data after the sample was modified by the X‐ray beam. Throughout the fitting steps, the BE and intensity of the main Ce^4+^ and Ce^3+^ peak were varied only, while all the other parameters were kept fixed. The atomic concentrations were obtained from the peak areas (including satellites) weighted by the relative sensitivity factors.

### Transmission Electron Microscopy

Preliminary TEM images, shown in Figure S6 of the Supporting Information, were collected through an FEI Tecnai G12 working with an accelerating voltage of 100 kV. HR‐TEM investigation was conducted using both TEM and STEM images acquired with a Thermo Fisher TALOS F200X G2 microscope, operating at an accelerating voltage of 200 kV. STEM mapping was performed using the 4 in‐column SSD Super‐X detectors.

### Raman Spectroscopy

Raman spectra were collected for each sample using a BWS465‐532H i‐Raman Plus (BWTEK – Metrohm Group Company), laser wavelength 532 nm, nominal power 30 mW. The relative power used for the spectra acquisition was 20% with an integration time of 720 s and mediated on three spectra for each sample.

### ICP‐MS

For the dissolution, the metallic components of the weighted samples were digested in aqua regia for 4 h at 100 °C and properly diluted. ICP‐MS for the determination of the Pt content was carried out with an Agilent Technologies 7700 × ICP‐MS (Agilent Technologies International Japan, Ltd., Tokyo, Japan). Sample digestion was carried out in aqua regia. The ICP‐MS instrument was equipped with an octopole collision cell operating in kinetic energy discrimination mode used for the removal of polyatomic interferences and argon‐based interferences.

## Conflict of Interest

The authors declare no conflict of interest.

## Supporting information



Supporting Information

## Data Availability

The data that support the findings of this study are available from the corresponding author upon reasonable request.
