# Supplementary material for: Pt NPs Supported on CeO2/C as Electrocatalysts for Oxygen Reduction Reaction: Novel Physicochemical Insights on the Synthesis and on the Improved Activity and Stability
Source: Small. 2025 May 5;21(25):2403127. doi: 10.1002/smll.202403127 (PMC12199119; doi:10.1002/smll.202403127)
Supplement: Supplementary file 1 — Supporting Information [file SMLL-21-2403127-s001.docx]

Supporting Information

Pt NPs supported on CeO_2_/C as electrocatalysts for oxygen reduction reaction: novel physicochemical insights on the synthesis and on the improved activity and stability.

Mattia Parnigotto,^a^ Gregorio Dal Sasso,^b^ Enrico Berretti,^c,d^ Marco Mazzucato,^a^ Federica Bertolotti,^e^ Alessandro Lavacchi,^c,d^ Maria Chiara Dalconi,^f^ Luca Gavioli, ^g^ and Christian Durante,^*,a^

*a) Department of Chemical Sciences, University of Padova, Via Marzolo 1, Padova, Italy*
*b) Italian National Research Council, Institute of Geosciences and Earth Resources, Via Gradenigo 6, Padova, Italy*

*c) Italian National Research Council, Institute of Chemistry of Organometallic Compounds, CNR-ICCOM, Via Madonna del Piano 10, Sesto Fiorentino, Florence, Italy*

d) *National Interuniversity Consortium of Materials Science and Technology, INSTM, Via G. Giusti 9, Florence, Italy*

e) *Department of Science and High Technology and To.Sca.Lab, University of Insubria, Via Valleggio 11, 22100 Como, Italy*

f) *Department of Geosciences, University of Padova, Via Gradenigo 6, Padova, Italy*

g) *Department of Mathematics and Physics, Università Cattolica del Sacro Cuore, via della Garzetta 46, Brescia, Italy*

**Table of contents**

**S1 WAXTS-DSE analysis of commercial ceria NPs** 3

**S2 Supporting Figures** 4

Figure S1WAXTS data of ceria NPs.

Figure S2 WAXTS-DSE analysis of PtCeO_2_(com)/C250 sample.

Figure S3 In-situ synchrotron solid state reactor.

Figure S4 Comparison of ceria Raman active mode

Figure S5 Atomic concentration for the ceria and Pt species.

Figure S6 TEM images and size distribution.

Figure S7 Electrochemical results comparison

Figure S8 CO stripping measures

Figure S9 Gas Diffusion Electrode cell.

Figure S10 Comparison of GDE results at ambient temperature in load cycle ADT protocol.

**S2 Supporting Tables 12**

Table S1 Results from the DSE analysis of CeO_2_ NPs

Table S2 Raman spectra carbon intensity band

Table S3 ECA H_UDP_ and ECA CO_stripping_

Table S4 GDE retain results

**S1 WAXTS-DSE analysis of commercial ceria NPs**

A separate WAXTS experiment was performed on commercial ceria NPs and experimental data was analysed through the DSE method. At first, the DSE modeling was based on a monovariate population of atomistic models of CeO_2_ nanocrystals that were built using the cubic (with unit cell parameter a = 5.415 Å) fluorite-like structure typical for ceria (space group $\text{Fm}\bar{\text{3}}\text{m}$). Structural and microstructural parameters as the lattice parameters, the average size and size dispersion of NPs, and the isotropic atomic displacement parameters were optimized by minimizing the differences between the experimental and computed X-ray scattering pattern. The most prominent misfit was observed on the 111 peak suggesting that a bivariate anisotropic description of ceria NP’s morphology could be more suitable (Figure S1a).^[38,39]^

Thus, a prismatic model was used, enabling the construction of a bivariate population of ceria NPs with two independent growth directions. The atomistic models of ceria NPs were built from the same structure but using a different unit cell setting; a primitive trigonal unit cell was obtained from the cubic one by the transformations: **a_t_** = (**−a_c_** + **b_c_**)/2; **b_t_**= (**−b_c_** + **c_c_**)/2; **c_t_** = **a_c_** + **b_c_** + **c_c,_** where t and c subscripts refer to the trigonal and cubic unit cell parameters, respectively.^[39]^ The cell parameters of the newly defined trigonal cell are a = 3.8290 Å and c = 9.3791 Å, with the c axis aligned to the [111] direction of the cubic system. Using this unit cell as a building block, a population of prismatic NPs was built according to two independent growth directions, one is the length along the c_t_ axis (*L*_c_) and one is the diameter of the circle of equivalent area in the a_t_b_t_ plane (*D*_ab_). Thanks to this approach, the DSE fit markedly improved, as shown in Figure S1b and highlighted by the goodness of fit (GoF) improving from 13.3 for the spherical model to 11.1 for the prismatic one.

**S2 Supporting Figures:**


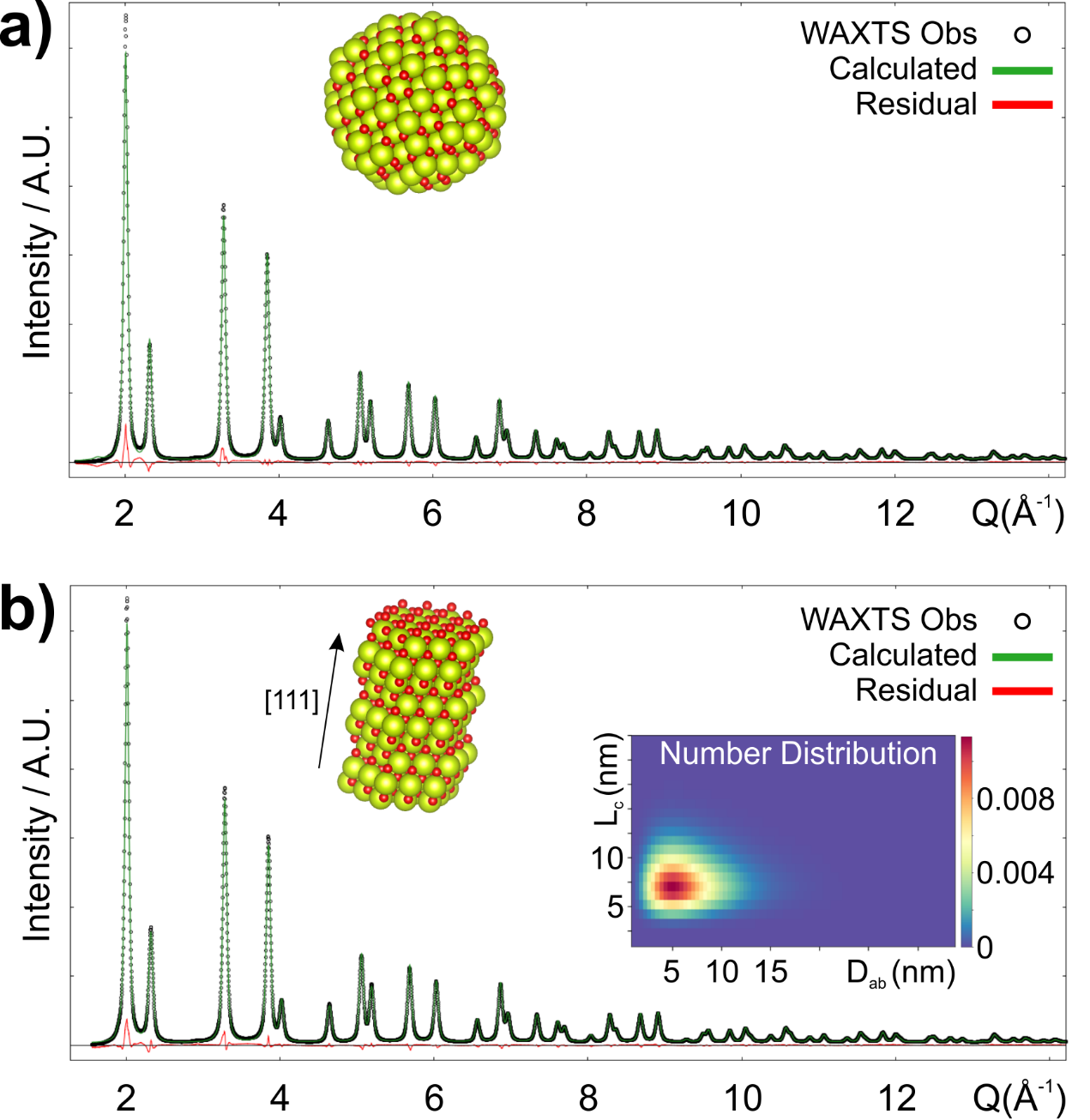


**Figure S1**. a) WAXTS data of ceria NPs (separately measured) and best DSE fit (green) obtained using a spherical model for ceria NPs; in the inset, atomistic model of CeO_2_ spherical NPs. b) WAXTS data of ceria NP and best DSE fit (green) obtained using a prismatic model for ceria NPs; in the inset, atomistic model of CeO_2_ NP constituting a bivariate population of atomistic models with two independent growth directions; a 2D map of the bivariate lognormal size distribution of CeO_2_ NPs, where the mass fraction is given in color code, is also reported; average D_ab_ and L_c_ are calculated from projections onto the corresponding axis.


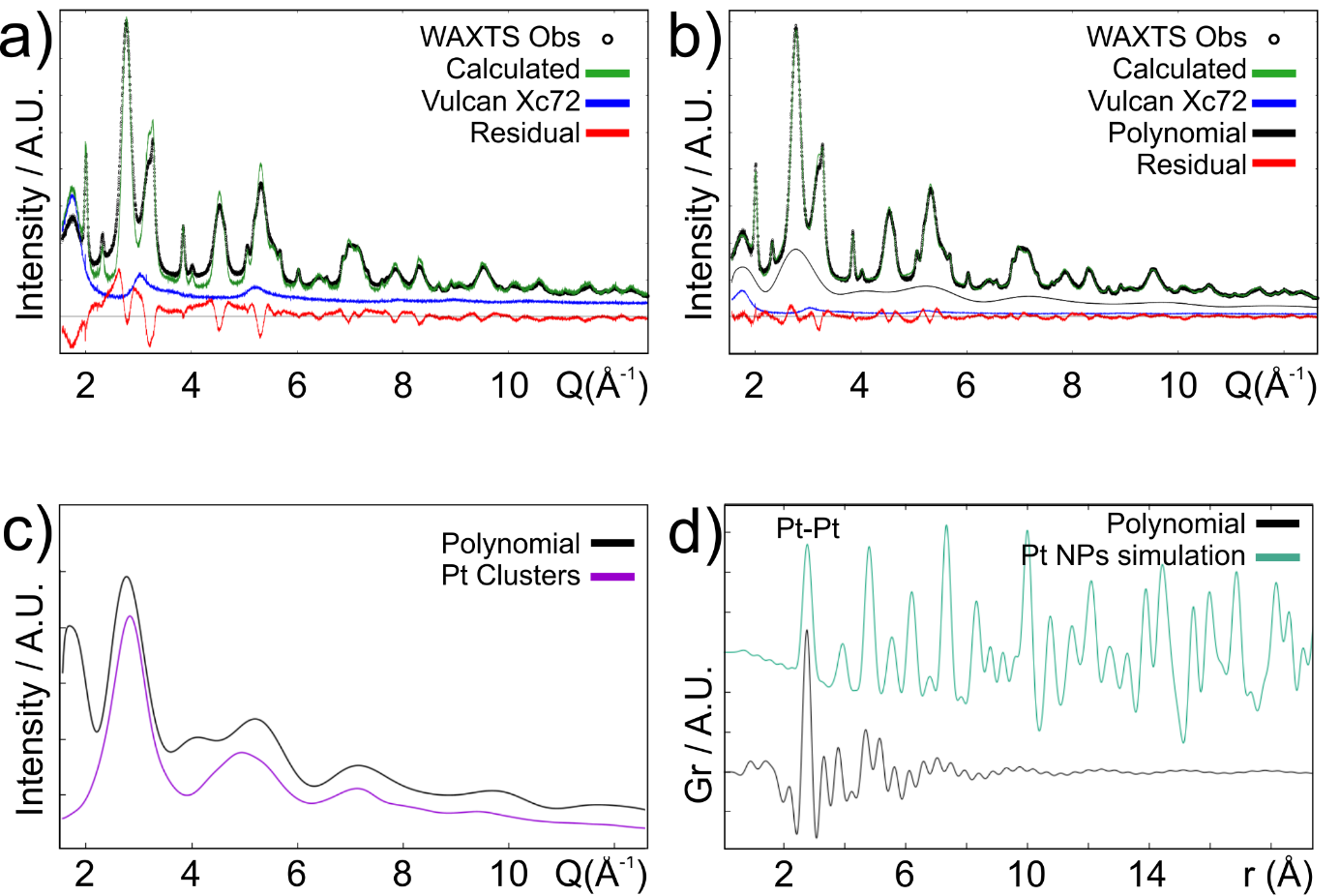


**Figure S2**: a) WAXTS-DSE analysis of PtCeO_2_(com)/C250 sample using a population of atomistic models of Pt NPs with the size and size dispersion resulting from the SAXS-DSE analysis; a significant contribution from diffuse scattering is missing. b) WAXTS-DSE best fit by introducing a polynomial function to the previous model. c) Comparison between the polynomial function introduced in (b) and the WAXTS-DSE simulation obtained from atomistic models of Pt sub-nanometric clusters. d) Comparison between the PDF of the polynomial function (black) used in (b) and the PDF simulated from the atomistic model of Pt NPs(green).


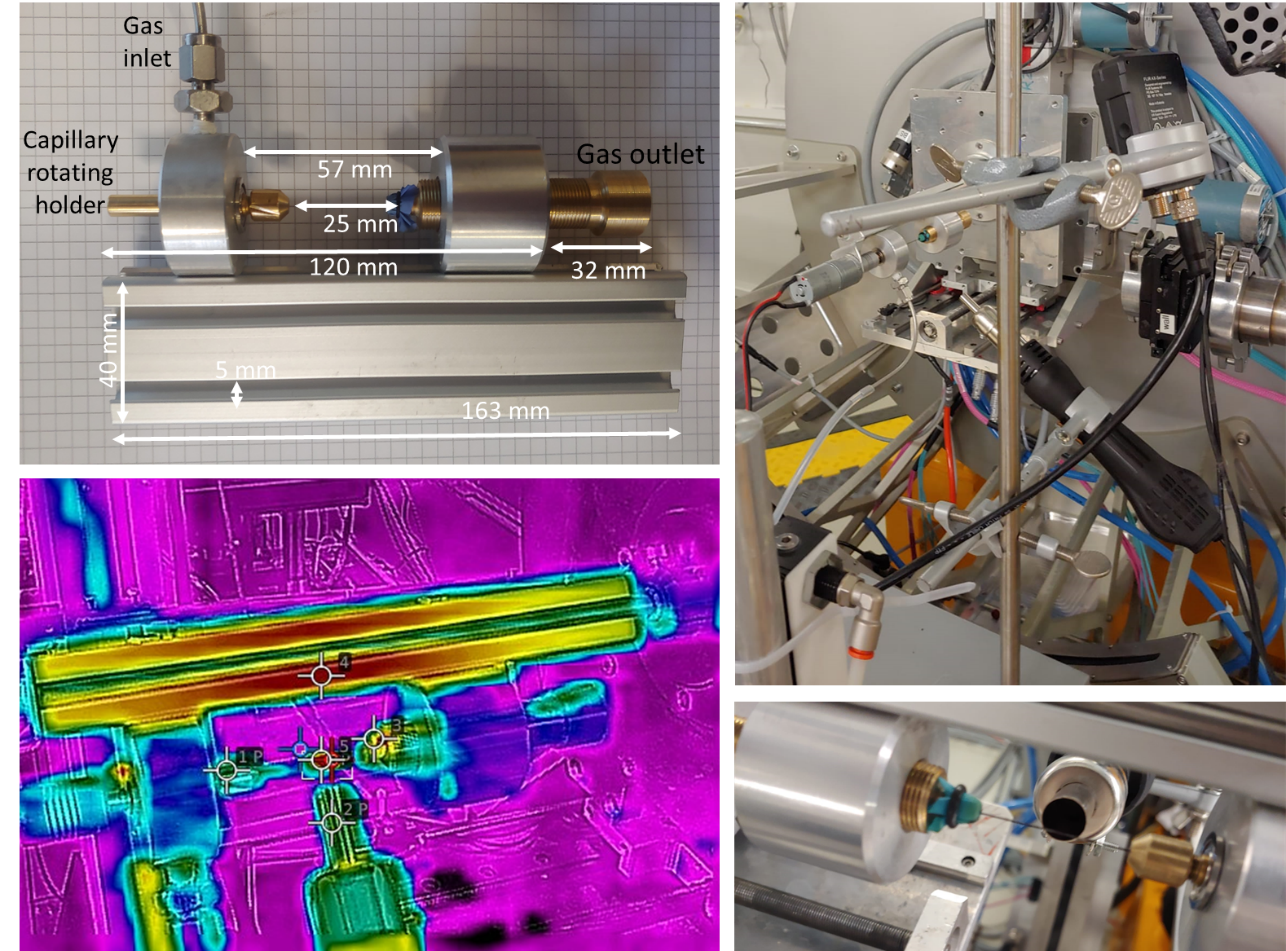


a)

c)

d)

b)

**Figure S3**: in-situ solid state reactor a) reactor dimensions; b) full XRD in-situ setup; c) thermal camera view and d) top-down image of the capillary and the hot gun.





**Figure S4**: Comparison of ceria Raman active mode between the PtCeO_2_/C250 with the PtCeO_2_(com)/C250.





**Figure S5**: Atomic concentration for the ceria and Pt species for each sample analyzed


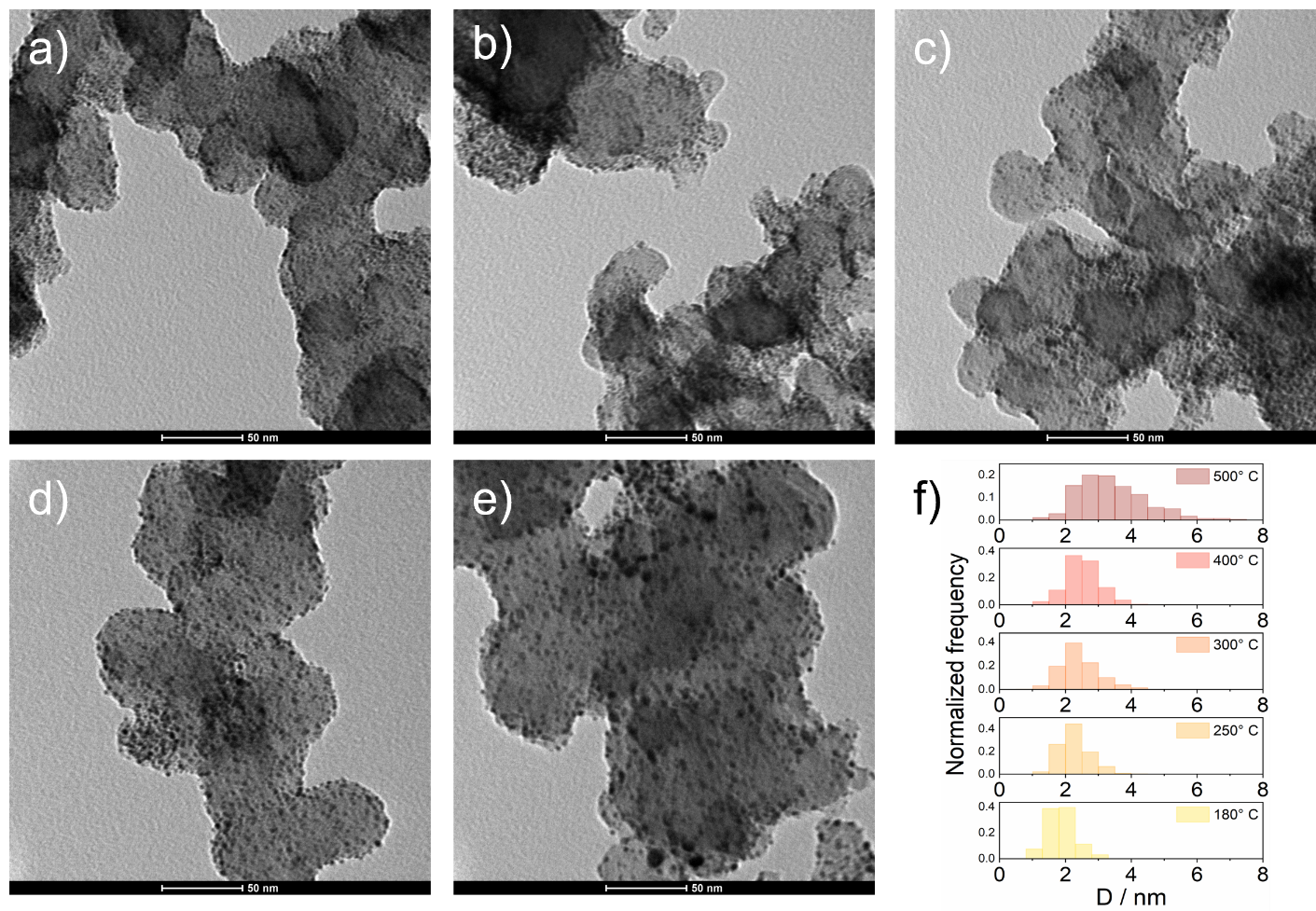


**Figure S6**: TEM images of a) PtCeO_2_/C 180°C; b) PtCeO_2_/C250; c) PtCeO_2_/C300; d) PtCeO_2_/C400; e) PtCeO_2_/C500; and f) size distribution obtained from 300 counts.





**Figure S7**: Electrochemical results in a heart cell configuration a) CV in argon atmosphere and b) LSV in oxygen atmosphere for the PtCeO_2_/C samples with 5% of CeO_2_ precursor at different temperatures of synthesis.





**Figure S8**: CO stripping measures for each analyzed sample a) PtCeO_2_/C180; b) PtCeO_2_/C250; c) PtCeO_2_(com)/C250; PtCeO_2_/C300; PtCeO_2_(10%)/C250; Pt/C250; PtCeO_2_/C400; PtCeO_2_/C500; TEC10E50E; and example of the saturation procedure, red line current evolution under the 0.05 V vs RHE applied voltage, change after atmosphere at 20 minutes.


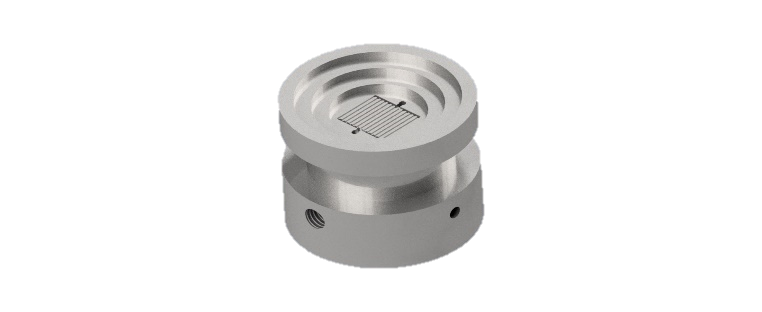


Carbon paper

Catalyst sprayed on the MPL of the GDL

Gas inlet (humidified)

Gas outlet

RE: RHE

CE: Platinum

Nafion membrane


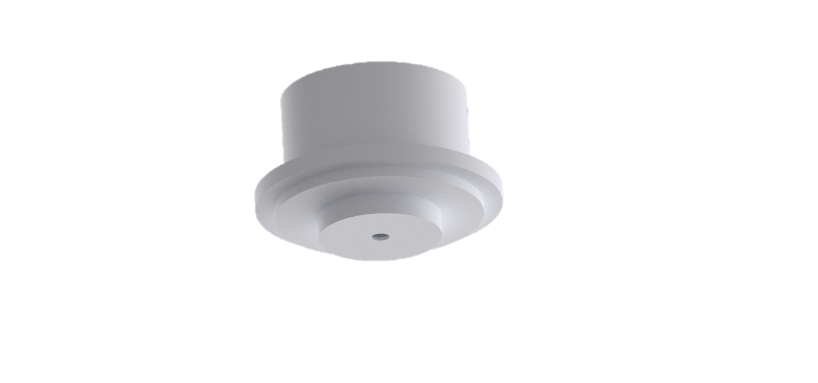


Carbon paper with pristine MPL as support for the deposited GDL

**Figure S9**: Gas Diffusion Electrode cell with a catalyst spray coated on the carbon paper with the MPL.


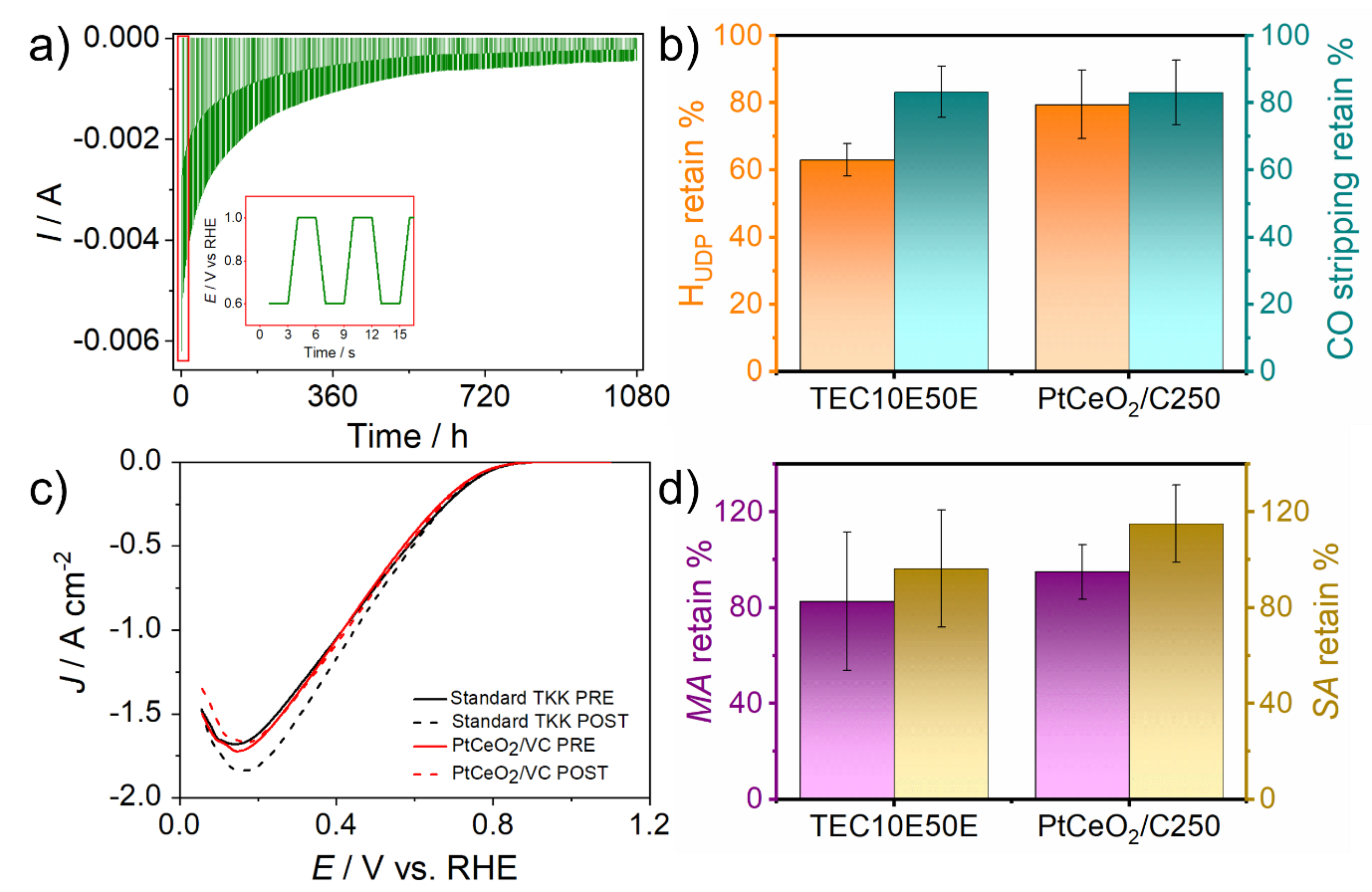


**Figure S10**: Comparison of GDE results at ambient temperature a) example of applied potential (in the red box) and developed current during the load cycle ADT protocol b) ECSA retains from CVs in Ar and CO stripping measures for the standard TEC10E50E and the PtCeO_2_/C250; c) LSV in O_2_ atmosphere in anodic direction at 100 mV s^−1^ for the standard TEC10E50E and the PtCeO_2_/C250; d) MA_0.65V_ and SA_0.65V_ retain for the standard TEC10E50E and the PtCeO_2_/C250.

**S3 Supporting Tables:**

Table S1: Results from the DSE analysis of CeO_2_ NPs using a bivariate population of prismatic atomistic models. Number- and mass-based average size (D_ab_, L_c_) of bivariate size distribution, their size dispersions (σ_Dab_, σ_Lc_), the refined cell parameter a (referring to the cubic unit cell), the isotropic atomic displacement parameters (B) of Ce and O atoms. The site occupancy factor for O atoms has been refined but no vacancies were detected.

| CeO_2_ NPs | *D*_ab_ (nm) | σ*D*_ab_ (nm) | *L*_c_ (nm) | σ*L*_c_ (nm) | a_c_ (Å) | B(Ce) (Å^2^) | B(O) (Å^2^) |
| --- | --- | --- | --- | --- | --- | --- | --- |
| Number-based distribution | 7.3 | 3.2 | 8.2 | 2.2 | 5.414 | 0.4399 | 0.8142 |
| Mass-based distribution | 10.5 | 4.5 | 8.8 | 2.4 |  |  |  |

Table S2: Parameters extrapolated from the deconvolution of the first-order Raman spectra of the different sample PtCeO_2_/C from 180 to 500 °C and with 5% or 10% of ceria. The parameters are compared with the Vulcan XC72 spectra.

|  | Raman shift / cm^−1^ | | | | | Normalized intensity | | | | | Normalized intensity ratio | | | |
| --- | --- | --- | --- | --- | --- | --- | --- | --- | --- | --- | --- | --- | --- | --- |
|  | D4 | D1 | D3 | G | D2 | I4 | I1 | I3 | IG | I2 | I_D4_/I_G_ | I_D1_/I_G_ | I_D3_/I_G_ | I_D1_/(I_D1_+I_G_) |
| Vulcan XC 72 | 1177 | 1349 | 1532 | 1591 | 1620 | 0.124 | 0.965 | 0.280 | 0.652 | 0.166 | 0.190 | 1.48 | 0.430 | 0.597 |
| PtCeO_2_/C180 | 1171 | 1349 | 1528 | 1591 | 1616 | 0.127 | 0.944 | 0.286 | 0.652 | 0.220 | 0.195 | 1.45 | 0.439 | 0.592 |
| PtCeO_2_/C250 | 1144 | 1349 | 1534 | 1592 | 1614 | 0.126 | 0.824 | 0.227 | 0.533 | 0.242 | 0.236 | 1.55 | 0.427 | 0.607 |
| PtCeO_2_/C300 | 1163 | 1350 | 1544 | 1590 | 1617 | 0.101 | 0.915 | 0.307 | 0.546 | 0.285 | 0.186 | 1.68 | 0.563 | 0.626 |
| PtCeO_2_/C400 | 1168 | 1350 | 1542 | 1592 | 1617 | 0.109 | 0.891 | 0.282 | 0.604 | 0.176 | 0.180 | 1.47 | 0.467 | 0.596 |
| PtCeO_2_/C500 | 1163 | 1349 | 1540 | 1589 | 1617 | 0.110 | 0.964 | 0.319 | 0.598 | 0.219 | 0.183 | 1.61 | 0.533 | 0.617 |
| PtCeO_2_(10%)/C 250 | 1169 | 1349 | 1532 | 1590 | 1616 | 0.110 | 0.959 | 0.292 | 0.623 | 0.219 | 0.176 | 1.54 | 0.469 | 0.606 |

Table S3: Electrochemical Area collected respectively from the H_UPD_ method and the CO_stripping_ method.

| Sample | ECA_HUPD_  (m^2^ g^−1^) | | ECA_CO_  (m^2^ g^−1^) | |
| --- | --- | --- | --- | --- |
| Pt/C250 | 60 ± 6 | | 76 ± 2 | |
| PtCeO_2_/C180 | 72 ± 12 | | 86 ± 3 | |
| PtCeO_2_/C250 | 76 ± 7 | | 95 ± 5 | |
| PtCeO_2_(10%)/C250 | 59 ± 9 | | 81 ± 3 | |
| PtCeO_2_(com)/C250 | 36 ± 4 | | 68 ± 3 | |
| PtCeO_2_/C300 | 74 ± 9 | | 90 ± 4 | |
| PtCeO_2_/C400 | 62 ± 6 | | 83 ± 3 | |
| PtCeO_2_/C500 | 55 ± 6 | | 76 ± 3 | |
| TEC10E50E |  | 81± 2 | |  |

Table S4: GDE retain results for the three tested samples, TEC10E50E; PtCeO_2_/C250 and Pt/C250.

|  | Retain % | | | |
| --- | --- | --- | --- | --- |
| Sample | ECA H_UPD_ | ECA CO_strip_ | MA | SA |
|  |  |  |  |  |
| TEC10E50E | 51 ± 7 | 64 ± 4 | 43 ± 5 | 62 ± 7 |
| PtCeO_2_/C250 | 45 ± 9 | 78 ± 6 | 62 ± 7 | 79 ± 9 |
| Pt/C250 | 52 ± 14 | 86 ±1 | 49 ± 9 | 56 ± 10 |
